# Whole Genome Analysis of 335 New Bacterial Species from Human Microbiota Reveals a Huge Reservoir of Transferable Antibiotic Resistance Determinants

**DOI:** 10.3390/ijms23042137

**Published:** 2022-02-15

**Authors:** Sami Khabthani, Jean-Marc Rolain, Vicky Merhej

**Affiliations:** 1 Faculté de Pharmacie, Aix-Marseille Université, 13005 Marseille, France; samikhabthani@outlook.fr (S.K.); jean-marc.rolain@univ-amu.fr (J.-M.R.); 2 IHU Méditerranée Infection, Institut de Recherche Pour le Développement (IRD), Assistance Publique-Hôpitaux de Marseille (AP-HM), Microbes Evolution Phylogeny and Infections (MEPHI), 19-21 Boulevard Jean Moulin, 13005 Marseille, France; 3 Faculté de Sciences Médicales et Paramédicales, Aix-Marseille Université, 13005 Marseille, France

**Keywords:** new bacterial species, human microbiota, antibiotic resistance, horizontal transfer, mobile elements

## Abstract

Background: The emergence and diffusion of strains of pathogenic bacteria resistant to antibiotics constitutes a real public health challenge. Antibiotic resistance genes (ARGs) can be carried by both pathogenic and non-pathogenic bacteria, including commensal bacteria from the human microbiota, which require special monitoring in the fight against antimicrobial resistance. Methods: We analyzed the proteomes of 335 new bacterial species from human microbiota to estimate its whole range of ARGs using the BLAST program against ARGs reference databases. Results: We found 278 bacteria that harbor a total of 883 potential ARGs with the following distribution: 264 macrolides-lincosamides-streptogramin, 195 aminoglycosides, 156 tetracyclines, 58 β-lactamases, 58 fosfomycin, 51 glycopeptides, 36 nitroimidazoles, 33 phenicols and 32 rifamycin. Furthermore, evolutionary analyses revealed the potential horizontal transfer with pathogenic bacteria involving mobile genetic elements such as transposase and plasmid. We identified many ARGs that may represent new variants in fosfomycin and β-lactams resistance. Conclusion: These findings show that new bacterial species from human microbiota should be considered as an important reservoir of ARGs that can be transferred to pathogenic bacteria. In vitro analyses of their phenotypic potential are required to improve our understanding of the functional role of this bacterial community in the development of antibiotic resistance.

## 1. Introduction

The discovery of antibiotics is one of the major medical breakthroughs of the 20th century, which significantly reduced morbidity and mortality due to bacterial infections [1]. However, the overall effectiveness of antibiotics is often compromised by the development of tolerance or resistance to these products [2]. The evolution of antibiotic resistance is complex, it frequently involves the occurrence and proliferation of gene mutations that confer resistance to one or many antibiotics [3]. The high frequency of an antibiotic-resistant mutants is maintained in the population when exposed to the antibiotic as a function of selective pressure [4]. Thus, the abuse and misuse of antibiotics have contributed to the more or less rapid appearance of antibiotic- resistant strains among medically important bacterial pathogens. Global action plans have been developed worldwide by the World Health Organization (WHO) to slow the emergence of antimicrobial resistance and reduce its spread. They aim to increase awareness of antimicrobial resistance and to encourage best use of antibiotherapy [5].

Yet, resistance to antimicrobials is a very old natural phenomenon that predates the use of antibiotics. Metagenomic studies of permafrost dating back 30,000 years have found antibiotic resistance genes (ARGs) against β-lactams, tetracycline and vancomycin [6]. ARG reservoirs have been found in all complex ecosystems [2]; they are as distributed as bacteria can be. The origin of ARGs and their diversity is due, among other things, to the interactions that can exist in a bacterial community [7]. Bacteria evolving in the same ecosystems interact with each other by chemical compounds, or by using specific secretion systems to ensure sustainability [8]. Thus, bacteria from human microbiota have been reported to synthesize antimicrobial substances [9]. In addition, *Staphylococcus lugdunensis*, isolated from the human nasal cavity, produces a compound called lugdunin, which demonstrates an activity against multidrug-resistant bacteria [10]. Indeed, many commonly used antibiotics are naturally synthesized compounds by microbes [11]. These natural antibiotics play an important role in the struggle for resources and the survival of the competing organisms [7]. Bacteria synthesizing antibiotics protect themselves against their own product by having ARGs, such as *Streptomyces cattleya* [12] and *Pseudomonas syringae* [13]. The prevalence of ARGs in microbial populations is but an adaptive response to challenges from compounds with antimicrobial activity, including the natural antibiotics [14].

Indeed, ARGs have evolved, diversified and spread long before the “antibiotic era”. Horizontal gene transfer (HGT) acts as a major driving force leading to the exchange of ARGs among diverse bacterial species, thus considerably fostering collaboration in bacterial population for the development of antibiotic resistance [15,16]. These transfers can be performed directly with free DNA (transformation) or through the involvement of vehicles such as plasmids (conjugation) or phages (transduction) [16]. In addition, genetic elements called “selfish genes” will allow, by mutualism, the adaptability of bacteria to toxic environments and favor their evolution in the ecosystem by acquisition of transposable elements such as transposons and plasmids containing ARGs [17]. ARG cassettes can be captured by integrons and introduced into bacterial genomes to confer antibiotic resistance to the host [18].

The HGT of ARGs is pervasive among bacteria, especially in the human-associated microbiota [19]. The great number of genomes, as much as 10^14^ bacterial cells, on one hand, and the close contact within the human body, particularly in the biofilm formation, on the other hand, greatly potentiate HGT [20,21]. Thus, the human microbiota is a breeding ground for bacterial warfare that harbors more than ten thousand biosynthetic gene clusters and genes encoding for ribosomally synthetized compounds with potential antimicrobial activity [22]. These findings from the application of omics technologies present the human microbiome as a new resource for finding new antibiotics. The downside is that the exposure to antibiotics promotes the development of resistance and enhances the transfer of ARGs. It has been shown that the diverse human microbiota, particularly the gut microbiota, represent an important reservoir for ARGs [23,24]. ARGs seem to be a feature of the human microbiome even without exposure to commercial antibiotics [25,26]. This shows the dynamism in the exchange of genetic material between species and raises the question of the still unknown wide range of resistance genes in the unknown species from human microbiota. A better estimation of the ARGs’ reservoir in the resident rather commensal bacteria would bring the issue of the ability of these ARGs to be transferred to bacteria of clinical interest.

Our study focused on the genome analysis of new bacterial species described for the first time in the human microbiota in the Institut Hospitalo-Universitaire Méditerranée Infection. These bacteria were isolated by culturomics, a novel approach that combines multiple culture conditions with rapid identification using MALDI- TOF mass spectrometry and 16S rRNA sequencing [27]. Culturomics enabled the isolation of bacterial species that have never been previously found in humans, thus expanding the repertoire of bacterial species in the human, and especially the gut, microbiota [28]. In addition, the next generation sequencing offered an unprecedented opportunity to explore the potential reservoir of ARGs in humans, investigate genetic variations and evaluate the HGT in bacteria from the human microbiota. In this study, we analyzed the genomes of 335 new bacterial species isolated from humans, mostly from feces samples, to look for the presence of ARGs using different computational approaches. The identified potential ARGs were subjected to further phylogenetic analysis, as well as an investigation of putative mobilizable elements.

## 2. Results

### 2.1. Prevalence and Description of ARGs Found in the New Bacterial Species from the Human Microbiota

**Figure 1:**
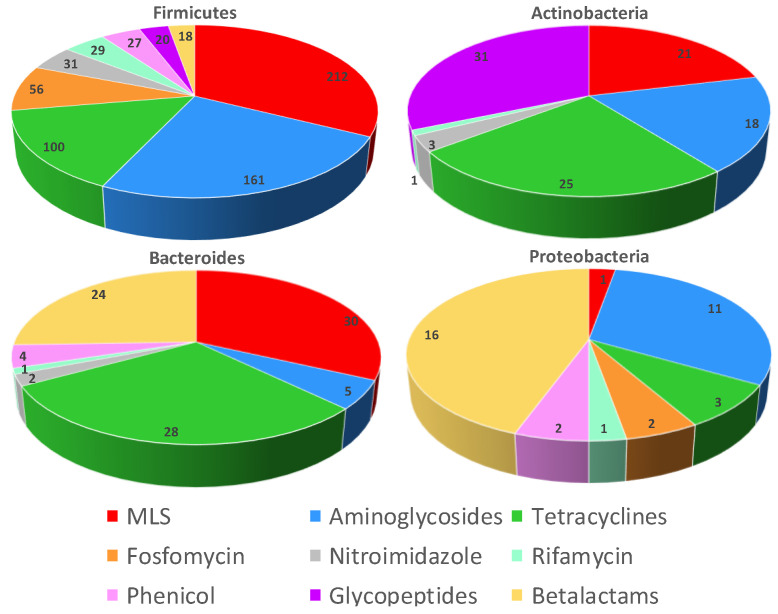
Graphical representation of the resistance gene distribution in the different phyla. The ARGs confer resistance to nine antibiotic families: macrolides-lincosamides-streptogramin (MLS) (264 ARGs), aminoglycosides (195), tetracyclines (156), β-lactams (58), fosfomycin (58), glycopeptides (51), nitroimidazoles (36), phenicols (33) and rifamycin (32 ARGs).

When using the reciprocal BLAST program, we found a total of 883 ARGs encoding for enzymes that may be involved in the development of antimicrobial resistance to the nine studied antibiotic classes (Figure 1). Details of all ARGs by classes found per bacteria is provided in (Supplementary Table S1). From the 335 studied bacteria, 278 have at least one ARG and 57 bacteria did not harbor ARGs (29 Firmicutes, 15 Actinobacteria, 7 Bacteroidetes/Chlorobi group, 3 Proteobacteria and 2 Fusobacteria) (Supplementary Table S1). The Firmicutes contained 654 ARGs that were found in 173 of the 203 analyzed genomes; they encode for ARGs that belong to the nine antibiotic classes. The most frequent ARGs were for Macrolides Lincosamides Streptogramins (MLS) (*n* = 212;32%), aminoglycosides (*n* = 161;25%) and tetracyclines (*n* = 100;15%) resistance genes (Figure 1). *Paenibacillus cagae* and *Paenibacillus ihumii* in the Firmicutes encoded the largest number of ARGs (*n* = 13). The most represented genes for Actinobacteria were vancomycin resistance genes in the family of glycopeptides (*n* = 31;31%), tetracyclines (*n* = 25;25%) and MLS (*n* = 21;21%); they were found in 58/73 studied genomes. Only six of the nine families of antibiotics were present, with a total of 99 resistance genes. For Bacteroidetes, 36 of 43 bacteria harbored at least one ARG, and we found 94 ARGs belonging to seven families of antibiotics, with the most common classes found for MLS (*n* = 30;32%), tetracyclines (*n* = 28;30%) and β-lactams (*n* = 24;26%). Finally, 11 of 14 bacteria from the Proteobacteria family harbored 36 ARGs from seven different families of antibiotics; the most common resistance genes were for β-lactam antibiotics (*n* = 16;44%), aminoglycosides (*n* = 11;31%) and tetracyclines (*n* = 3;8%) (Figure 1).

The most frequent ARGs were those against MLS (a total of 264 ARGs, 30%), aminoglycosides (*n* = 195;22%), tetracyclines (*n* = 156;17.5%), fosfomycin (58;7%) and β-lactams (58;7%) (Figure 1 and Supplementary Table S1). These gene families represented 83% of the total of ARGs detected (Figure 1). Antibiotic-modifying enzymes accounted for the highest number of ARGs (61%). They included esterases, transferases, lyases and phosphorylases for MLS. N-acetyl, O-nucleotidyl and O-phosphotransferases were found for aminoglycosides, oxidoreductases for tetracyclines and β-lactamases for β-lactams. Other mechanisms involving enzymes for target protection such as rRNA methylases were also found and can be responsible for resistance to aminoglycosides and MLS (Table 1). The most represented enzymes of these families were by far the inactivating enzyme Tet (22%), followed by the transferases Llm (13%), Aac (12%) and Vat (9%) and the methylases Erm (9%).

### 2.2. New Putative ARGs Conferring Resistance to β-Lactams, Fosfomycin and Vancomycin

**Figure 2:**
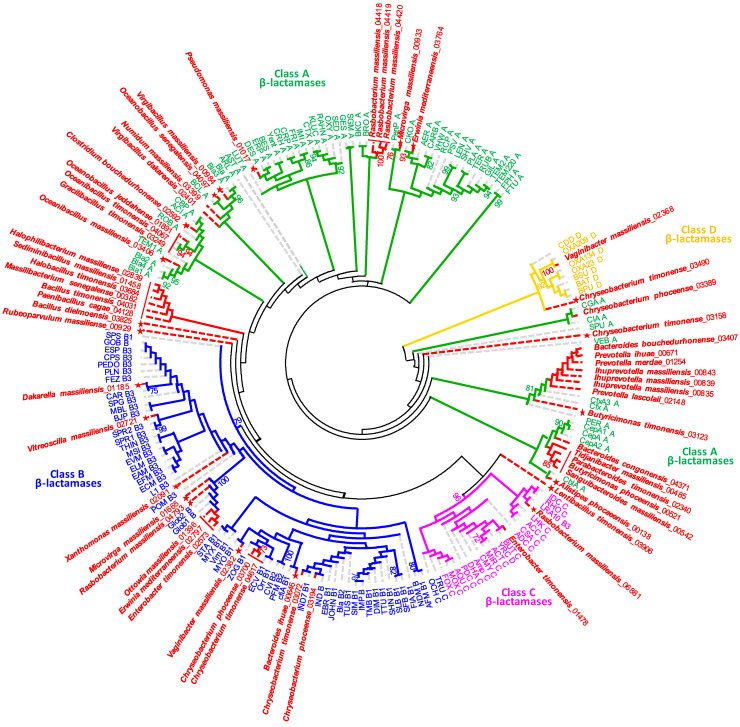
Phylogenetic tree based on β-lactamase sequences. Sequences were aligned based on their amino acid sequences and phylogeny was inferred using the maximum likelihood method. The percentage of trees in which the associated taxa clustered together (100 bootstrap replicates) is shown next to the branches. New ARGs found in the studied species are colored in red, stars indicate a new variant or new ARG-based one and the bars indicate a new family of ARG.

The protein sequences of ARGs potentially conferring β-lactam resistance were aligned with the already known β-lactamase domains in the Conserved Domain Database (CDD) [29]. We searched for motifs, i.e., sequence patterns that occur repeatedly, in these proteins by MAST scanning within the MEME suite [30]. This structural and functional analysis showed that 44 sequences conserved at least two amino-acid motifs, allowing its classification as the serine β-lactamase, including 41 in class A, two in class C and one in class D (Table 2). We identified fourteen ARGs encoding for potential amino acid bonds to zinc molecules that can be considered as metallo-β-lactamase, including six in the subclass B1, seven in the subclass B3 and one with the HARLDQ motif (Figure 2, Table 2). The phylogenetic tree based on the analysis of β-lactams-resistance proteins showed that fifteen proteins from twelve species represented a single line descent together with already known ARGs for β-lactams, including the Cfx, CepA, CGA, CIA, Bla and BCL in class A, to IND in class B, to Amp in class C and OXA in class D. The twelve species correspond to *Ihuprevotella* sp., *Prevotella* sp., *Bacteroides bouchedurhonense*, *Bacteroides congonensis*, *Chryseobacterium phoceense*, *Virgibacillus dakarensis*, *Numidum massiliensis*, *Chryseobacterium* sp., *Enterobacter timonensis* and *Vaginibacter massiliensis*. The pairwise comparison of sequence identity of all sequences generally confirmed the phylogenetic grouping, as revealed in Figure 2 and Table S2.

The fifteen protein sequences yielded high similarity values of at least 70% with their closest phylogenetic relatives. In the phylogenetic tree, seven other species grouped weakly with already known ARGs for β-lactams, but their sequence identity was less than 70 (Table S2), while 36 proteins from 30 species occupied a well-separated position. Among these, nineteen species (21 ARGs) were distributed into six distinct clusters (containing at least two proteins). They yielded sequence similarity was well under 70% and therefore could not be assigned to known ARGs for β-lactams. On the basis of sequence analysis and phylogeny, these proteins can be considered as new putative β-lactamase variants, including twenty-nine in class A, thirteen in class B and *Rasbobacterium massiliensis* 06,881 in class C. The well-separated clusters of proteins can be attributed to new putative β-lactamase families, of which four are in class A and two in class B.

**Figure 3:**
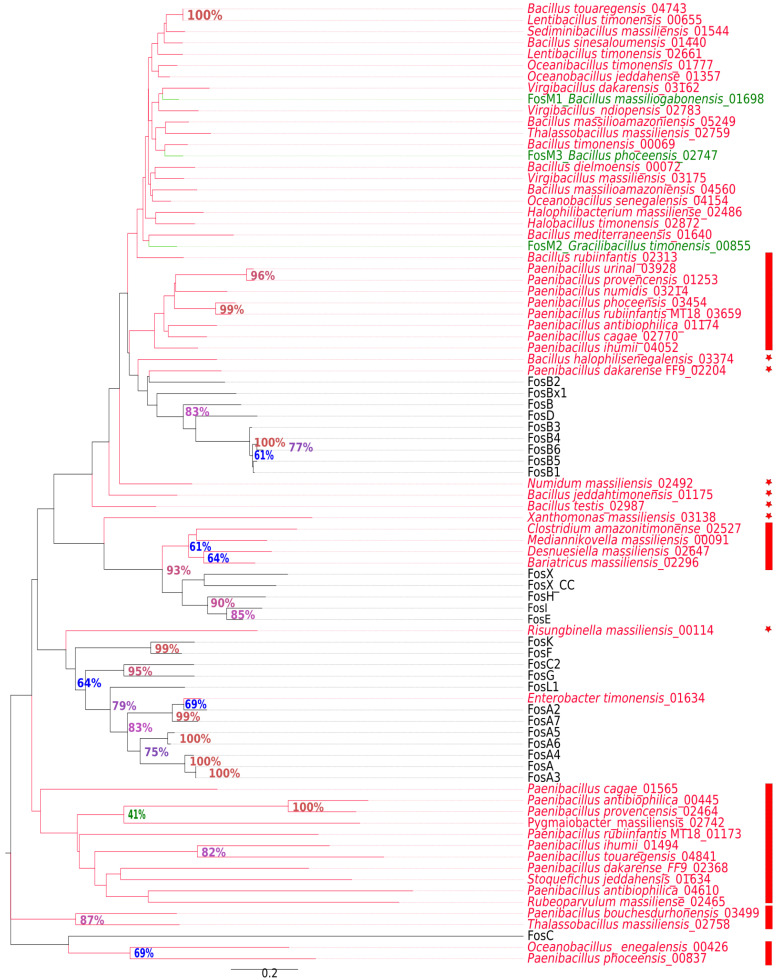
Phylogenetic tree based on Fosfomycin protein sequences. New ARGs found in the studied species are colored in red, stars indicate a new variant or new ARG-based one and a vertical bar indicates a new family of ARG. Green lines indicate genes that have been previously characterized biologically as fosfomycin resistance genes [32].

The alignment of ARG sequences potentially conferring resistance to Fosfomycin with protein domains within CDD showed that the 58 putative Fos proteins rather belonged to the large family of metallothiol transferases in the superfamily of vicinal oxygen chelate (VOC). Similar to other metalloenzymes in this family (FosA, FosB and FosX), these Fos proteins render fosfomycin inactive by opening the oxirane [31]. These genes are distributed in two phyla: 56 in Firmicutes and two in Proteobacteria. In Firmicutes, *Paenibacillus antibioticophila* contains three *fos* genes alone, ten bacterial species have two and the remaining have only one (Figure 3). The phylogenetic tree based on the analysis of fosfomycin proteins showed that five proteins represent a well-separated branch. The pairwise comparison of sequence alignment showed that these sequences have less than 64% identity with their closest phylogenetic relatives already known to confer fosfomycin resistance (Table S3). While the most divergent known metallo-transferases leading to resistance to fosfomycin has a 66% similarity with already known FosB, these five proteins may be representative of new distinct variants of fosfomycin resistance (Figure 3). They included two genes from *Bacillus halophilisenegalensis* (03374) and *Paenibacillus dakarense* (02204) that seem to be new variants of the *fosB* family; *Numidum massiliensis*, *Bacillus Jeddahtimonensis* and *Bacillus testis* seem to be a novel class of ARGs closely related to the *fosB/D/M* family. *Xanthomonas massiliensis* (03138) and *Risungbinella massiliensis* (00114) formed a phylogenetic branch that was distinct from all known fosfomycin resistance proteins, and their similarities with all analyzed sequences were slightly below the putative class delineation threshold of 59% defined herein. Therefore, these proteins seem to represent novel ARGs conferring resistance to fosfomycin.

**Figure 4:**
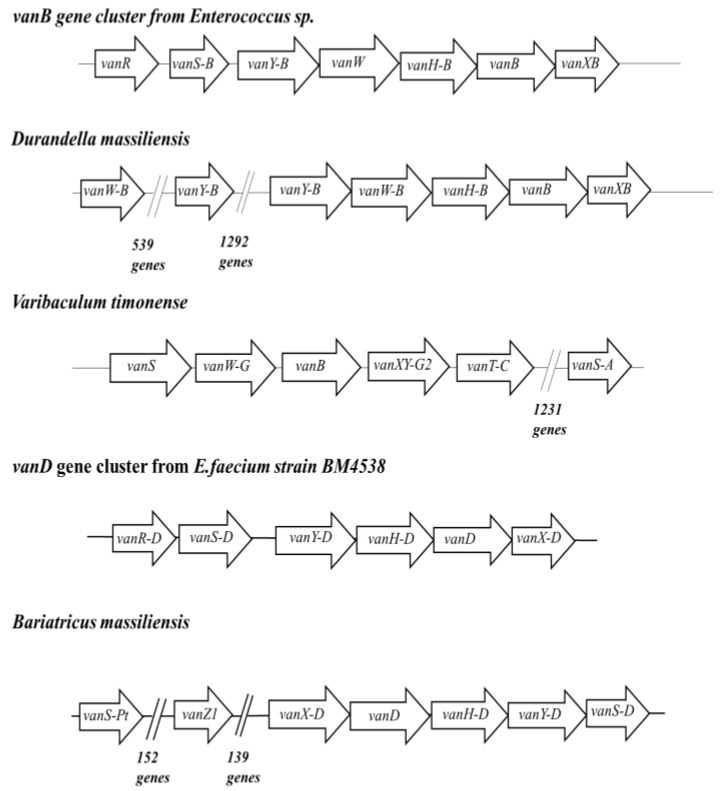
Schematic representation of the clusters of glycopeptide resistance genes found in three bacteria from the human gut in comparison with *vanB* and *vanD* gene clusters in *Enteroccoccus* sp.

Finally, vanB and vanD glycopeptide resistance genes were found in only five studied bacteria. When considering the ordering of the functionally significant elements from the vancomycin resistant operon in the region of interest which contains the identified *vanB* and *vanD* genes, we could find that three of these five bacteria have at least four other genes related to the vancomycin resistance gene operon (Figure 4). Indeed, *Durandella massiliensis, Varibaculum timonensis* and *Bariatricus massiliensis* were found to harbor in the neighborhood around *vanB* or *vanD*, genes that are homologous (100% identity and coverage) to genes that are usually associated with the operon of the vancomycin resistance gene. The genomic syntheny of the regions containing *van* resistance genes with *vanB* and *vanD* gene clusters from vancomycin-resistant enterococci (VRE) (Figure 4) suggest that the three clusters identified herein can mediate resistance to glycopeptides. Similar to other *vanB* clusters, they may induce the production of peptidoglycan precursors terminating in d-alanyl-d-lactate (d-Ala-d-Lac) instead of d-alanyl-d-alanine (d-Ala-d-Ala), which drastically decreases the affinity for vancomycin. It is noteworthy that *vanB*-type genes have a moderate level of resistance to vancomycin compared with *vanA* genes.

### 2.3. Origin of ARGs in the Human Microbiota

**Figure 5:**
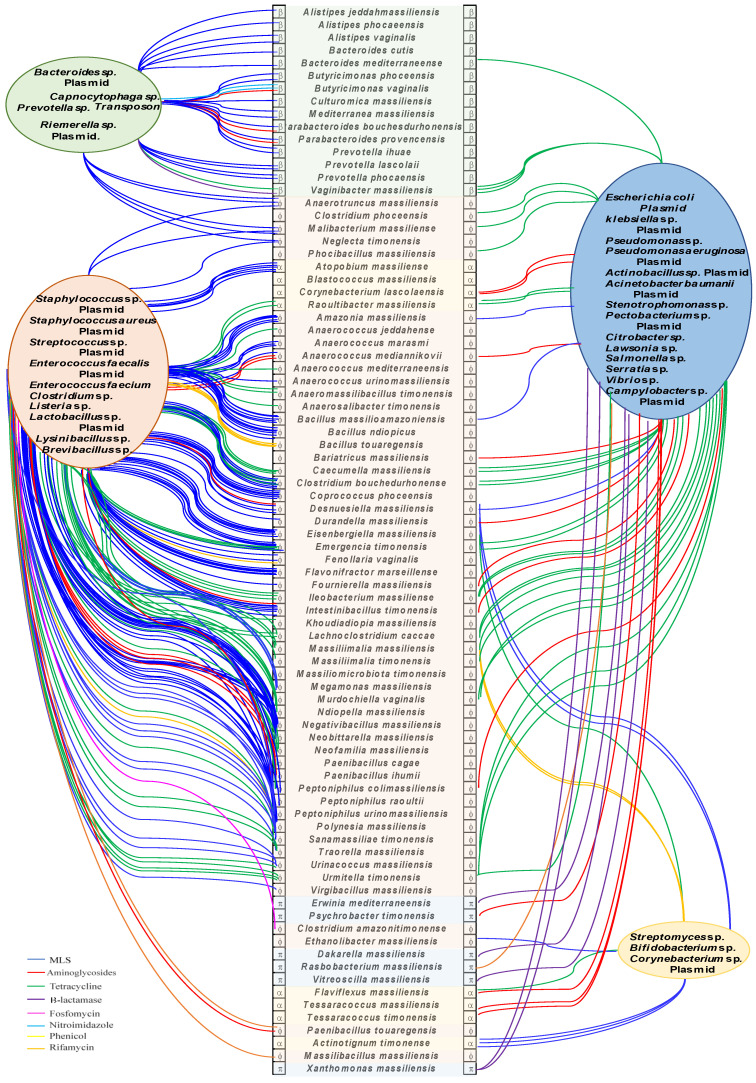
The melting-pot inside the human microbiota. Network showing possible horizontal transfers between the new species analyzed herein and commensal and pathogenic species from *Bacteroidetes* (β)*, Firmicutes* (ϕ)*, Proteobacteria* (π) and *Actinobacteria* (α). The edges were colored according to the antibiotic families.

To investigate the human microbiota as a melting-pot for the horizontal transfer of ARGs, we performed an evolutionary analysis based on ARGs phylogenies, as well as analysis of the genetic environment context and GC content of genes versus genomes (Figure S1). The phylogenies showing the evolutionary history of the ARGs revealed the putative involvement of 111 ARGs in gene exchange between species from human microbiota and human commensal or pathogenic bacteria based on biosafety level ≥ 2 (https://bacdive.dsmz.de accessed on 15 April 2021) (Supplementary Figures S2–S19). Indeed, these trees constructed on the basis of gene sequences showed bacteria from human microbiota with potential ARGs identified herein in a robust cluster (bootstrap higher than 70) with species from a distinct phylum. Among these ARGs, 49 were to MLS, 28 to tetracyclines, 20 to aminoglycosides, five to phenicol, five to β-lactams, two to rifamycin, one to nitroimidazole and one to fosfomycin (Figure 5). Phylogenies showed sixteen of these robust chimeric nodes involving *Staphylococcus aureus* and its three plasmids and 29 nodes with *Enterococcus* sp. and its three plasmids. *Escherichia coli* and its plasmid and *Pseudomonas aeruginosa* and its plasmid were observed in four chimeric nodes each. Finally, phylogenies showed robust grouping between bacteria from human microbiota and plasmids from *Klebsiella* sp. (3 nodes) and one plasmid from *Acinetobacter baumannii* (one node) (Figure 5). Bacteria from human microbiota found in these chimeric nodes belonged to the Firmicutes, Bacteroides/Chlorobi, Actinobacteria and to Proteobacteria in 77, 20, 8 and 6, respectively, of the 111 ARGs. *Megamonas massiliensis* harbored the greatest number, as many as four potentially transferred resistance genes, followed by *Anaerococcus mediannikovii, Paenibacillus cagae*, *Peptoniphilus colimassiliensis*, *Polynesia massiliensis* and *Vaginibacter massiliensis* with three genes. The GC content of these genes differed significantly from the genomic average and reached values greater than 10 in 47 genes. Interestingly, these potentially transferred ARGs were located next to each other in the genome, which could form a probable resistance island that can be transferred *“en bloc”* to other organisms (Table 3).

**Figure 6:**
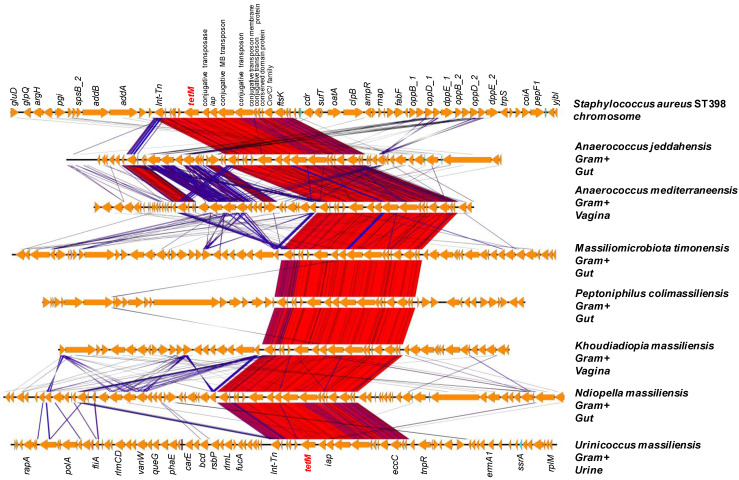
Comparison of the cluster gene surrounded by an integrase and transposase containing *tetM ARG found in Staphylococcus aureus* ST398 (Accession number: AM990992.1) isolate in a case of human endocarditis and seven new bacterial species described in this work. The figure illustrates potential gene transfer between bacteria isolated from human microbiota. The color gradient from blue to red indicates the percentage of sequence similarity which was calculated via tblastx with the Easyfig tool [33] between the genes in the genetic environments studied.

The search for mobile genetic elements revealed the presence of 61 transposases and 20 possible integrated plasmids in the studied bacteria coding for ARGs (Table 3). The phylogenetic trees showed robust groupings between a human microbiota species and a pathogenic bacterium from the same or different phyla (Supplementary Figures S2–S19). Transposases were found in the genomes of thirteen species that formed a robust phylogenetic group with pathogenic bacteria in the ARG protein-based trees (Table 3). These mobile genetic elements were located in the region coding for the ARGs (not more than ten genes up and down the gene) which suggests possible gene acquisition by transposition. Thus, a gene cluster surrounded by an integrase and a transposase containing a tet(M) gene (Figure 6) found on *Staphylococcus aureus* ST398 (Accession number: AM990992.1) chromosome was found in eight Gram+ bacteria analyzed, five of which were isolated from the gut, two from the vagina and one from urine (*Anaerococcus jeddahensis*, *Anaerococcus mediterraneensis*, *Ndiopella massiliensis*, *Massiliomicrobiota massiliensis*, *Polynesia massiliensis*, *Peptoniphilus colimassiliensis*, *Khoudiadiopa massiliensis* and *Urinacoccus massiliensis*) (Figure 6 and Figure S8, Table 3). A plasmid search in these bacteria showed that seven bacteria had a repUS43 plasmid on the same contig as the *tet(M)* gene. The *S. aureus* genome showed an origin of replication CAQ49392.1 described as transcriptional regulator, Cro/CI family with a replication initiation domain (Figure 6).

**Figure 7:**
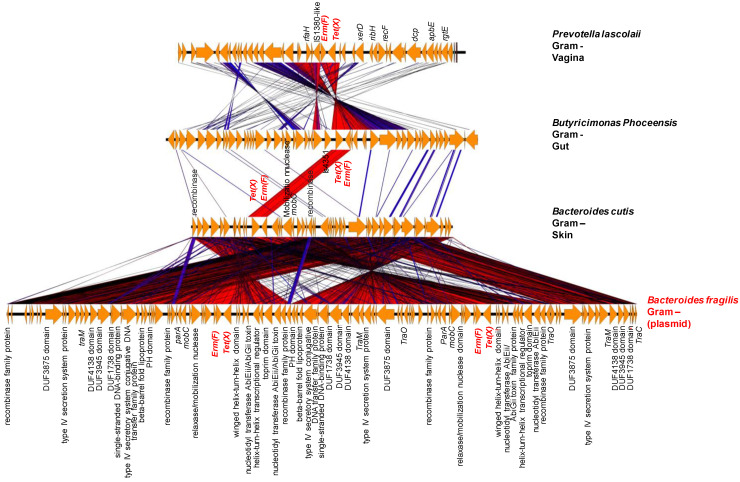
Comparison of the genetic environment from *Bacteroides fragilis strain FDAARGOS* (Accession number: CP054002) plasmid containing *erm(F)* and *tet(X)* ARGs isolated from the transverse colon and three new species studied. The figure illustrates potential gene transfer between bacteria isolated from human microbiota. The color gradient from blue to red indicates the percentage of sequence similarity between the genes in the genetic environments studied.

**Figure 8:**
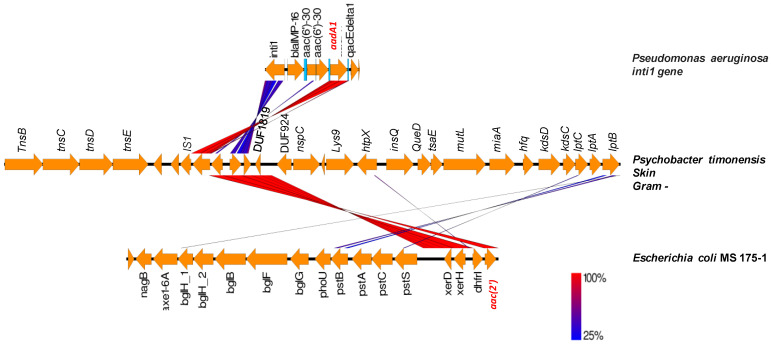
Comparison of the genetic environment of *Psychrobacter timonensis*, integron from *Pseudomonas aeruginosa* (Accession number: AJ584652) containing *aadA1* ARG and *Escherichia coli* MS 175-1 with *aac(2′)* ARG (Accession number: ADUB00000000). The figure illustrates potential gene transfer between bacteria isolated from human microbiota. The color gradient from blue to red indicates the percentage of sequence similarity between the genes in the genetic environments studied.

Another example includes *Bacteroides cutis*, *Butyricimonas phoceensis* and *Prevotella lascolaii* that harbored two genes, *ermF* and *tetX,* next to each other that were found to be similar to genes on a plasmid from *Bacteroides fragilis* strain FDAARGOS (Accession number CP054002) (Figure 7). The representation in Figure 7 shows a very high similarity of the genes in the genetic environment of these two genes with a plasmid from *Bacteroides fragilis*. *Bacteroides cutis* does not contain a plasmid according to PlasmidFinder; however, some elements of plasmid mobility such as the *mobC* gene as well as recombinases and conjugative transposon in the Tra family were found in the environment of these genes. These ARGs from *Bacteroid cutis* may be the result of lateral transfer; they may have been conveyed by a plasmid that integrated into the genomes of this bacterium. We found a transposase IS4351 followed by *tet(X)* and *erm(F)* in *Butyricimonas phoceensis* genome, whereas *Prevotella lascolaii* has in 5′ an insertion sequence (IS1380) followed by an *erm(F)* and *tet(X)*. These data suggest that these ARGs can be mobilizable. Likewise, *Psychrobacter timonensis* has two aminoglycoside resistance genes, *aadA1* and *aac(2′)*, next to transposases (Figure 8). The *aadA1* gene has already been characterized in an Int1 integron of *Pseudomonas aeruginosa* (Genbank: AJ584652.2) isolated from a lower respiratory tract infection and the following *aac* gene in an *Escherichia coli* MS 175-1 from the human gut. The two genes *aadA1* and *aac2* are surrounded by an insertion sequence and two tyrosine recombinases in *Psychrobacter massiliensis* and *E. coli* genomes.

## 3. Discussion

The present work is the first description of the resistome of a large collection of new bacterial species from the human microbiota. Our results reveal the presence of 883 ARGs in the 335 analyzed bacterial genomes. The most prevalent ARGs were encoding for MLS, aminoglycosides, tetracyclines, β-lactams and fosfomycin, which is in agreement with the results reported in various studies on the human gut https://resistomap.datalaboratory.ru/ accessed on 15 May 2021, knowing that 77% of our studied bacteria were isolated from the gut [34,35].

Many ARGs identified herein can be considered as putative new variants, new ARGs and even new ARG families (Figure 2 and Figure 3 and Supplementary Figures S2–S19). They represent well-separated novel sequences within the ARGs and could be distinguished from their nearest phylogenetic relatives through molecular analysis, in particular the lack of sequences similarity (under 64% for β-lactamase and 70% for fosfomycin resistance). The sequence similarity threshold that can be used to define the novelty of protein sequence is relatively dependable. Indeed, New Delhi Metallo β-lactamase 21 (NDM21) was described as a new variant, even though it has 99.9% nucleotide sequence similarity with NDM5 [36]. Concerning the *fos* genes, *fosB4*, *fosB5* and *fosB6* have been defined as subtypes of the *fosB* genes with protein sequence similarities with FosB1 of 99.3%, 99.3% and 97.8%, respectively [37]. In a previous work, we described a new resistance gene family *fosM*, consisting of three genes *fosM1*, *fosM2* and *fosM3* detected in *Bacillus massiliogabonensis*, *Gracilibacillus timonensis* and *Bacillus phoceensis*, respectively, and which have protein sequence similarities of less than 70% with the referenced *fos* genes. Thus, we confirmed the functional activity of such genes by in vitro techniques [32]. Likewise, Sommer et al., (2009) characterized antibiotic resistance genes in human microflora and obtained an average of 69.5% nucleotide coverage with GenBank sequences [38]. Altogether, these results demonstrate the great diversity of resistance mechanisms and that the genes known so far in the bibliography and referenced are only a tiny part of the repertoire of ARGs existing in nature [39].

We found evidence of putative transfers of ARGs between the studied bacteria and pathogenic bacteria. Phylogenetic trees with known ARGs and the hits resulting from ARG blasts highlighted some potential HGTs with pathogenic bacteria. The difference between the GC content of ARGs and their host genome indicate possible lateral acquisition. We estimated the transferability of these ARGs and their mechanism of integration into these new bacterial species genomes through the presence of transposon and plasmids. Thus, we provide some examples of potential exchanges of ARGs by transposition in chromosomes or plasmids between our studied species from different body sites and pathogenic bacteria such as *S. aureus*, *B. fragilis*, *P. aeruginosa* and *E. coli* (Figure 6, Figure 7 and Figure 8 ). These examples do not determine the direction of the ARG exchange; it is possible that these bacteria can be a source of dissemination of ARGs into pathogenic bacteria. It has already been shown in vitro that gut commensal bacteria of the genus Bacteroides and Clostridiales can transfer ARGs to pathogenic bacteria [40,41,42,43]. The comparison of metagenomically assembled genomes from fecal samples with samples collected 10 years apart for each of two participants, using high-throughput chromosomal conformation capture, estimated as much as twelve HGT per year on average [44].

This work shows that the new bacterial species isolated from human microbiota could be progenitors of new ARGs that may disseminate in bacteria of clinical interest. Likewise, *Moraxella* sp. chromosomal sequences was shown to be a probable reservoir that provides colistin resistance mcr-like genes [45]. Genomic analysis of 64,628 Gram-negative bacteria [46] demonstrated a wide distribution of *mcr-1* homologues, with over 13,658 BLAST hits. In the same work, the rhizome of *mcr-1* showed that *Moraxella pluranimalium* was the putative progenitor of this gene, while the BLAST shows, with very high confidence (criteria identity ≥ 90% and alignment ≥ 98%), that 6% of the *E.coli* analyzed possess *mcr-1*. This therefore implies the wide distribution by HGT of this gene, unknown before 2015 [47]. Another example is the chimeric ARG New Delhi Metallo β-lactamase (NDM-1), resulting from the fusion of the first six amino acids of the aminoglycoside ARG (aphA6) and a metallo β-lactamase (MBL) that most likely occurred in *Acinetobacter baumannii* [48]. This gene, discovered in 2008, has spread very rapidly and alarmingly in various Gram-negative pathogenic bacteria around the world [49]. Altogether, our findings reveal the genetic diversity of ARGs and draw attention to the potential role of human microbiota in the current and future antimicrobial resistance threats.

It is noteworthy that our results found herein do not represent a general view of the frequency of ARGs in all body sites, as there was a selection bias and a predominance of bacteria from the gut. The majority of bacteria studied were Firmicutes, which may influence the predominance of some ARG families over others. Taken together, these biases are due to the themes of the work carried out within our IHU team, which is more focused on the culture of bacteria from the gut. Additionally, we do not have information on antibiotic usage, which would be interesting to understand the evolutionary history of ARGs. Nevertheless, the main limitation of this work remains in the in silico method. We applied a computational approach to assess the antimicrobial resistance within a complex environment such as the human microbiota searching for ARGs on the basis of enzyme homology. Even though the protein sequences were very close or identical to already known active ARGs responsible for clinical antibiotic resistance, these in silico results need in vitro confirmation. Future experimental research that characterizes the antimicrobial profile of these ARGs in human pathogens and commensals would be very useful to provide clinical evidence.

## 4. Materials and Methods

### 4.1. Materials

In this study, we analyzed the genome sequences of 335 new bacterial species that have been isolated from human microbiota for the first time in the Institut Hospitalo-Universitaire Méditerranée Infection using culturomics [28] (Supplementary Table S1). The studied bacteria were identified in different biological specimens, specifically in stool (76%), vaginal swabs (9%), urine (4%), skin swabs (3%) and sputum and nasal swab (3%) samples. They were distributed as follows: 203 Firmicutes, 73 Actinobacteria, 43 Bacteroidetes, 14 Proteobacteria and 2 Fusobacteria. The genomes of these bacteria were previously sequenced using different whole genome sequencing (WGS) strategies and reads were assembled by the IHU bioinformatic team. Genome sequences have been deposited in the database of the European Bioinformatics Institute (EMBL-EBI) https://www.ebi.ac.uk/genomes/bacteria.html accessed on 15 December 2020. We retrieved the genome sequences and annotated them ab initio with the PROKKA1.12 pipeline [50].

### 4.2. Computational Method for Predicting ARGs

Genome-based ARG tracking was performed by using a BLAST-based approach to query input amino acid sequence data from the 335 bacteria for the presence of a pre-determined set of ARG determinants contained in AMR, CARD and ARG-ANNOT reference databases [51,52,53]. The output was sorted to keep the hits corresponding to enzymatic mechanisms of resistance, i.e., inactivating genes or target-site alteration. Hits obtained with a minimum threshold of 50% identity and 70% coverage were considered as significant. Protein sequences corresponding to the obtained hits from this first BlastP were individually compared with all proteins in each bacterial genome by reciprocal BlastP. The best BLAST for each protein was extracted using a Perl script. The best hits for each resistance gene-bacteria and bacteria-resistance gene pairs were compared in order to determine the number of reciprocal best hits for each pairwise comparison. The number of reciprocal best hits was counted using an expectation value (E) of <10^−4^ as the stringency threshold for determining a valid best hit.

### 4.3. Phylogenetic Analysis

The potential ARGs were used to construct individual protein trees. The amino acid sequences were retrieved from the different databases, aligned using Clustal [54] and further optimized visually. Maximum likelihood (ML) analyses were conducted in the MEGA v7 program with default settings [55]. Clade robustness was assessed using a bootstrap analysis with 1000 replicates [56]. We used Bioedit [57] to calculate the identity matrix of the alignments of fosfomycin and β-lactamase amino acid sequences. This allowed us to determine the rate of similarity between well-described reference sequences in order to set the parameters for defining new ARGs or new variants. A clustering of two or more new ARGs can be considered as putative new resistance gene families.

### 4.4. Bioinformatic Characterization of Conserved Protein Domains and Motifs

The conserved domain database, CDD [29] (https://www.ncbi.nlm.nih.gov/Structure/cdd/cdd.shtml accessed on 15 March 2021), was used to find the protein domains in order to characterize the enzyme functional class. The MEME/MAST Suite was used to identify the patterns/motifs which are specific to a particular family or subfamily of β-lactamases [30] (http://meme.nbcr.net accessed on 15 May 2021). The conserved patterns were derived using the pattern-search tool with default parameters of fingerprint width of 10 residues and minimum occurrence of two patterns per sequence. Motif scanning was performed by the Motif Alignment and Search Tool (MAST) [30]. The determination of family specific patterns/motifs helps to assign the newly identified β-lactamases to one or the other family or subfamily; it can be very useful to reveal gene novelty. The genomic context of potential vancomycin resistance genes was examined looking for specific genetic organization and the presence of an operon. The BLAST comparisons of the genomic region containing the potential ARGs to genomic sequences from NCBI database allowed the search for regions with similarly annotated content.

### 4.5. Detection of Mobile Genetic Elements Associated with ARGs and Determination of GC Content

Further in silico analysis of the genetic context of the predicted ARGs was performed to search for the presence of mobile elements. Transposases were searched up to ten genes upstream and downstream for the predicted ARGs using a locally developed program that considered the annotation of the genome to locate the positions of potential mobile genetic elements. The plasmids were searched within the PlasmidFinder database [58] with a minimum threshold of 90% identity and 90% coverage. The sequence of the ARGs and its corresponding genomes in FASTA format were used to calculate the GC%. The start and end codons were not deleted from the sequence for all the tested genes. Comparative analysis between the studied genomes containing ARGs and genomes of pathogenic bacteria with homologs to these ARGs was generated and drawn using Easyfig software [33].

## 5. Conclusions

We conducted an exhaustive in silico analysis in order to describe the resistome of new bacterial species from the human microbiota. We have shown that these new bacterial species may have interacted with pathogenic bacteria and exchanged ARGs in one way or another. We also highlighted potential new genes together with possible new resistance mechanisms. These putative new ARGs may be transferred to transient clinical bacteria associated with human infection. Knowledge of the human microbiota and its ARG reservoir is important for understanding the long-term future challenges of antibiotic resistance.

## Figures and Tables

**Table 1:** Distribution and diversity of resistance genes found by BlastP listed by antibiotic families.

ATB	Genes Detected by BlastP	Diversity	Total
Aminoglycosides	*aac(2′)-IIb,aac(3),aac(3)-If,aac(3)-Ih,aac(3)-IIe,aac(3)-IIIa,aac(3)-IIIb,aac(3)-VIII,aac(3)-Xa,aac(3)-XI,aac(6′),aac(6′)-34,aac(6′)-35,aac(6′)-Iad,aac(6′)-Iz,aacC7,aacC9,AAD(3′’),aad9,aadA1,aadA13,aadA31,aadD1,aadE,aadK,aadS,ant(3′’)-IIa,ant(3′’)-IIb,ant(3′’)-IIc,ant(4′)-Ic,ant(6),ant(6)-Ia,ant(6)-Ib,ant(6)-Ic,ant(9),aph,aph(2′’)-IIa,aph(3′)-Ia,aph(3′)-IIa,aph(3′)-IIc,aph(3′)-IIIa,aph(3′)-IVa,aph(3′)-Va,APH(6),aph(6)-Ia,aph(6)-Id,aph(9)-Ia,aac(2′).*	53	195
Betalactams	*bla,bla1,blaBCL-1,blaBKC,blaBKC-1,blaCAR-1,blaCBP,blaCIA-1,blaCIA-4,blaCKO-1,blaCMY-100,blaIND-4,blaIND-7,blaLRA-10_like,blaLUT-5,blaMYO-1,blaOXA-209,blaP,blaPOM,blaSPR-1,blaSPU-1,blaZOG-1,CblA,cepA,cepA-49,cfiA,cfxA,cfxA3,TEM.*	29	58
Fosfomycin	*fos,fos_related,fosA,fosA2,fosA-491618165,fosA7,fosA8,fosB,fosB2,fosB-38141535,fosBx1,fosX.*	12	58
Glycopeptides	*vanB,vanC1,vanD.*	3	51
MLS	*chrB_rRNA_meth,ereD,erm(31),erm(32),erm(35),erm(46),erm(49),erm(A),erm(B),erm(D),erm(F),erm(G),erm(X),erm(Y),ermk,llmA,lnu(A),lnu(AN2),lnu(C),lnu(D),lnu(G),lnuE,mph(B),mphI,mphK/ycbJ,mphL,mphM,mphO,myrA,rlmA,vgb(B),vgbC,cfr,cfr(B),cfr-Cb,clbC,vat,vat(B),vat(F),vat(H),vatI.*	41	270
Nitroimidazole	*nimA,nimB,nimD,nimE,nimJ.*	5	36
Phenicol	*cat,cat86,catA1,catA13,catA15,catA16,catA4,CatB,catB3,catP,catV,cpt.*	12	33
Rifamycin	*arr-269927220,arr-Ms,rphB,rphC,rphD.*	5	32
Tetracyclines	*otr(A),tet(32),tet(36),tet(M),tet(O),tet(Q),tet(T),tet(W),tet(X),tetB(P).*	10	156
	Total	170	889

**Table 2:** ARGs with probable β-lactamase activity and the analysis of the conserved motifs to determine their nature, either serine (class A, C and D) or metallo (class B).

Class	Conserved Motifs				Bacterial Species (ARGs ID)
A	STFK	SDN	EIDLN	KTG	*Rubeoparvulum massiliensis (00929)*
	STFK	SDN	EPDLN	KSG	*Gracilibacillus timonensis (03249), Oceanibacillus timonensis (04067)*
	STFK	SDN	EPDLN	KTG	*Rasbobactérium massiliensis (04418)*
	STFK	SDN	ETDLN	KSG	*Oceanobacillus jeddahense (01891)*
	STFK	SDN	ETDLN	KTG	*Microvirga massiliensis (00933)*
	STFK	SDN	ETELN	KTG	*Pseudomonas massiliensis (01017)*
	STFK	SDN	EVELN	KSG	*Lentibacillus timonensis (03906)*
	STFK	SDS	X	KTG	*Rabobacterium massiliensis (04420)*
	STHK	SDN	EPALN	KTG	*Virgibacillus massiliensis (00984)*
	STHK	SDN	EPELN	KSG	*Virgibacillus dakarensis (02401)*
	STHK	SDN	EPELN	KTG	*Numidum massiliensis (03399), Oceanobacillus senegalensis (04097)*
	STHK	SDN		KSG	*Erwinia mediterraneensis (03764)*
	STVK	SDS	X	KTG	*Rasbobacterium massiliensis (04419)*
	STYK	SDN	EPDLN	KSG	*Bacillus timonensis (04031)*
	STYK	SDN	EPDLN	KSG	*Massilibacterium senegalense (00382), Oceanbacillus massiliensis (03406)*
	STYK	SDN	EPELN	KSG	*Halobacillus timonensis (03684)*
	STYK	SDN	EPELN	KSG	*Paenibacillus cagae (04128)*
	STYK	SDN	EPNLN	KSG	*Halophilibacterium massiliense (02839)*
	STYK	SDN	ETELN	KSG	*Bacillus dielmoensis (03825), Clostridium bouchedurhonensis (02592)*
	STYK	X	ETELN	KSG	*Sediminibacillus massiliensis (01458)*
	SVFK	SDN	X	KTG	*Alistipes phocaeensis (00138), Bacteroides bouchedurhonensis (03407), Bacteroides congolense (04371), Butyricimonas phoceensis (00521), Butyricimonas timonensis (03123), Chryseobacterium phoceense (03389), Ihuprevotella massiliensis (00839), Ihuprevotella massiliensis (00843), Ihuprevotella massiliensis (00835), Parabacteroides timonensis (02340), Prevotella ihumii (00671), Prevotella lascolaii (02148), Prevotella merdae (01254), Sanguibacter massiliensis (00542), Tidjanibacter massiliensis (00485)*
	SVFK	SDN	X	X	*Chryseobacterium timonensis (03158), Chryseobacterium timonense (03490)*
B	HARLDQ				*Vitreoscilla massiliensis (02721)*
B1	HXHXD				*Bacteroides ihumii (00646), Chryseobacterium phoceensis (03194), Chryseobacterium phoceensis (03700), Chryseobacterium timonensis (03072), Chryseobacterium timonensis (04617), Vaginibacter massiliensis (02382)*
B3	HAHADH				*Xanthomonas massiliensis (02091)*
	HGHFDH				*Dakarella massiliensis (01185)*
	HXHXDH				*Enterobacter timonensis (02573), Erwinia mediterraneensi (02797), Microvirga massiliensis (01665), Ottowia massiliensis (01396), Rasbobacterium massiliensis (04793)*
C	SVSK	YAN		KTG	*Enterobacter timonensis (01478)*
	SVSK	YSN		KTG	*Rasbobacterium massiliensis (06881)*
D	STFK	SCV	X	KTG	*Vaginibacter massiliensis (02368)*

**Table 3:** ARGs found in the genomes of human microbiota that can be issued from lateral transfer according to the closely related species found in robust phylogenies, the difference between their GC content and the mean GC% of the genomes (Dif) and the presence of transposases nearby. Species in red indicate the possible *en bloc* transfer. * indicates prophage integrase and plasmid recombination enzyme five proteins upstream or downstream of the specific protein.

Genome	Gene	ARGs	Closely Related Species	[Dif]	Transposase
*Anaerococcus jeddahense*	01594	*tet(M)*	*Staphylococcus aureus*	5	01589
*Massiliomicrobiota timonensis*	00843	*tet(M)*		0	0838
*Peptoniphilus colimassiliensis*	00489	*tet(M)*		14	00484
*Khoudiadiopia massiliensis*	00250	*tet(M)*		5	00245
*Ndiopella massiliensis*	00481	*tet(M)*		11	00476
*Urinicoccus massiliensis*	01024	*tet(M)*		0	01019
*Polynesia massiliensis*	06038	*tet(M)*		11	06033
*Anaerococcus mediterraneense*	01722	*tet(M)*		1	01727
*Anaerococcus mediannikovii*	00814	*ant*	*Streptococcus mitis*	5	00817_00819
*Anaerococcus mediannikovii*	00808	*erm*	*Streptococus pyogenes*	3	00817_00819
*Anaerococcus mediannikovii*	00812	*aph*	Plasmid in *Enterococcus faecalis*, plasmid in *K. oxytoca*	11	00817_00819
*Intestinibacillus timonensis*	00006	*aph*		8	
*Peptoniphilus colimassiliensis*	00331	*aph*		5	00324_00326
*Peptoniphilus colimassiliensis*	00335	*erm*	*S. aureus, S. pyogenes,S. parasanguis*	20	00324_00326
*Peptoniphilus raoultii*	01230	*erm*		5	01231_01233
*Urinacoccus massiliensis*	01047	*erm*		6	01037
*Clostridium bouchedurhonense*	00004	*erm*	*Enterococcus faecium/Staphylococcus epidermidis,* plasmid in *S. pyogenes*, plasmid in *streptococcus sanguis*, plasmid in *Lactobacillus reuteri, Clostridioides difficile*	13	
*Emergencia timonensis*	01552	*erm*		16	01558
*Negativibacillus massiliensis*	02069	*erm*		16	02058
*Intestinibacillus timonensis*	01053	*erm*		20	
*Sanamassiliae timonensis*	00487	*erm*		16	00477
*Neobittarella massiliensis*	00011	*erm*		24	
*Psychrobacter timonensis*	02207	*aac*	*Escherichia coli*	5	02201
*Desnuesiella massiliensis*	01710	*ant*	*S. aureus*	5	01706
*Malibacterium massiliense*	00202	*tet*	*Lawsonia intracellularis*	10	
*Phocibacillus massiliensis*	01825 *	*tet*		5	
*Clostridium phoceensis*	03063 *	*tet*		11	
*Megasphaera vaginalis*	01258	*tet*	*Clostridiodes difficile*	14	
*Emergencia timonensis*	03403	*tet*	*Vibrio sp., E. faecalis Clostridium septicum*	12	03398
*Bariatricus massiliensis*	00013 *	*aad*	Plasmid in *Campylobacter jejuni*	9	
*Neglecta timonensis*	01846	*erm*	*Lysinibacillus sphaericus,Bacteroides thetaiotaomicron,Bacterioides ovatus*	26	01848_01850
*Megamonas massiliensis*	00045	*lnu*	*Streptococcus agalactiae*	2	
*Megamonas massiliensis*	00845	*lnu*		2	
*Megamonas massiliensis*	01002	*lnu*		2	
*Megamonas massiliensis*	01006	*lnu*		2	

## Data Availability

Not applicable.

## Supplementary Materials

The following supporting information can be downloaded at: https://www.mdpi.com/article/10.3390/ijms23042137/s1.
