# Supplementary material for: Whole Genome Analysis of 335 New Bacterial Species from Human Microbiota Reveals a Huge Reservoir of Transferable Antibiotic Resistance Determinants
Source: Int J Mol Sci. 2022 Feb 15;23(4):2137. doi: 10.3390/ijms23042137 (PMC8874588; doi:10.3390/ijms23042137)
Supplement: Supplementary file 1 [file ijms-23-02137-s001.zip › Supplementary files/Supplementary tables and figures_ revised.pdf]

Table S2: Similarity matrix containing the 58 potential beta-lactamases detected in this study and their best blast hits. Values marked in bold represent the highest percentage identity obtained when compared to all know beta-lactamases.

Sequences	Amp	BCL	BKC	Bla1	Bla2	Bla3	Bla	Bla4	CAR3	CblA	CBP	CepA1	CepA2	CepA	cfiA1	Cfx	CfxA3	CGA	CIA	CKO	Glob1	Glob2	IND	IND7	LRA10	LUT	MYO	OXA209	PenP	POM	SPR1	SPR2	SPU	TEM1	ZOG1
Alistipes phocaeensis 00138	7%	18%	19%	16%	15%	17%	17%	16%	4%	**46%**	19%	38%	38%	38%	6%	24%	24%	31%	33%	16%	4%	4%	4%	4%	6%	16%	6%	6%	20%	5%	6%	6%	32%	21%	5%
Bacillus dielmoensis 03825	7%	49%	30%	60%	61%	53%	53%	**61%**	7%	17%	46%	16%	16%	16%	9%	16%	16%	18%	18%	31%	4%	5%	6%	6%	6%	40%	7%	8%	36%	7%	7%	7%	16%	57%	6%
Bacillus timonensis 04031	6%	51%	33%	**56%**	**56%**	48%	50%	**56%**	7%	18%	43%	18%	18%	18%	8%	17%	17%	19%	18%	32%	4%	5%	5%	5%	7%	39%	7%	9%	33%	7%	8%	8%	17%	55%	5%
Bacteroides bouchedurhonense 03407	9%	15%	15%	14%	14%	15%	15%	15%	6%	24%	15%	33%	33%	33%	7%	**83%**	**83%**	24%	24%	15%	4%	3%	5%	6%	8%	20%	4%	7%	19%	6%	4%	4%	26%	16%	6%
Bacteroides congonensis 04371	9%	20%	21%	18%	19%	19%	20%	18%	4%	40%	19%	73%	**74%**	73%	7%	35%	35%	36%	38%	16%	5%	5%	3%	3%	7%	22%	4%	8%	20%	7%	5%	5%	37%	20%	5%
Bacteroides ihuae 00646	6%	5%	6%	7%	8%	6%	6%	7%	7%	6%	8%	7%	7%	7%	**53%**	6%	6%	9%	8%	7%	8%	7%	27%	27%	5%	7%	29%	5%	6%	8%	9%	9%	8%	6%	32%
Butyricimonas phoceensis 00521	6%	13%	18%	16%	17%	15%	16%	16%	3%	34%	17%	**48%**	**48%**	**48%**	6%	34%	34%	30%	29%	13%	5%	5%	4%	4%	6%	17%	5%	8%	17%	5%	6%	6%	31%	17%	5%
Butyricimonas timonensis 03123	8%	17%	16%	12%	13%	15%	16%	13%	5%	25%	17%	35%	35%	35%	6%	**67%**	**67%**	26%	27%	17%	5%	4%	5%	5%	8%	21%	5%	8%	18%	6%	5%	5%	28%	17%	5%
Chryseobacterium phoceense 03194	4%	7%	6%	7%	6%	8%	8%	7%	8%	3%	5%	3%	3%	3%	24%	4%	4%	6%	5%	7%	7%	7%	**88%**	76%	3%	5%	24%	5%	5%	9%	7%	7%	5%	5%	25%
Chryseobacterium phoceense 03389	7%	18%	17%	19%	19%	21%	20%	19%	3%	35%	23%	32%	32%	32%	7%	25%	25%	**73%**	72%	17%	7%	6%	6%	7%	6%	17%	7%	9%	19%	6%	5%	5%	50%	22%	6%
Chryseobacterium phoceense 03700	5%	6%	4%	6%	7%	5%	5%	6%	8%	6%	8%	4%	4%	4%	33%	5%	5%	6%	5%	4%	8%	6%	26%	25%	3%	6%	**47%**	5%	4%	9%	6%	6%	5%	6%	**47%**
Chryseobacterium timonense 03072	4%	7%	5%	6%	5%	8%	8%	6%	7%	4%	5%	5%	5%	5%	26%	6%	6%	6%	6%	6%	8%	7%	72%	**95%**	4%	6%	24%	5%	5%	8%	8%	8%	6%	5%	24%
Chryseobacterium timonense 03158	7%	18%	17%	17%	17%	19%	19%	17%	4%	29%	19%	33%	33%	33%	7%	25%	25%	44%	43%	17%	5%	5%	5%	7%	6%	20%	6%	9%	21%	5%	4%	4%	**47%**	20%	6%
Chryseobacterium timonense 03490	8%	16%	17%	18%	17%	18%	18%	18%	2%	35%	20%	31%	31%	31%	8%	23%	23%	66%	**67%**	16%	5%	6%	4%	6%	7%	16%	6%	9%	19%	6%	5%	5%	50%	20%	5%
Chryseobacterium timonense 04617	4%	6%	5%	5%	6%	6%	5%	5%	8%	5%	7%	5%	5%	5%	31%	3%	3%	7%	6%	5%	8%	7%	25%	24%	4%	5%	**49%**	6%	4%	9%	8%	8%	6%	5%	46%
Clostridium bouchedurhonense 02592	5%	49%	30%	47%	47%	45%	45%	47%	6%	17%	**59%**	18%	18%	18%	8%	15%	15%	20%	19%	28%	3%	5%	3%	4%	6%	35%	6%	9%	32%	5%	6%	6%	18%	48%	4%
Dakarella massiliensis 01185	5%	6%	6%	6%	6%	6%	6%	6%	**48%**	5%	6%	7%	7%	7%	8%	3%	3%	4%	4%	5%	11%	11%	10%	9%	4%	6%	8%	3%	5%	19%	18%	18%	3%	6%	8%
Enterobacter timonensis 01478	**74%**	7%	9%	6%	7%	8%	8%	6%	6%	7%	6%	7%	7%	7%	5%	9%	9%	7%	7%	9%	3%	3%	3%	4%	41%	7%	4%	4%	8%	8%	5%	5%	7%	6%	4%
Enterobacter timonensis 02573	4%	4%	4%	4%	5%	6%	6%	5%	11%	4%	5%	3%	3%	3%	7%	5%	5%	4%	5%	6%	**54%**	50%	8%	7%	3%	5%	7%	3%	5%	13%	12%	12%	4%	4%	8%
Erwinia mediterraneensis 02797	5%	5%	5%	6%	7%	7%	7%	6%	11%	4%	6%	4%	4%	4%	8%	6%	6%	6%	6%	7%	**52%**	49%	7%	8%	4%	5%	5%	2%	6%	13%	12%	12%	5%	5%	8%
Erwinia mediterraneensis 03764	8%	32%	33%	35%	36%	31%	31%	35%	5%	18%	27%	17%	17%	17%	6%	14%	14%	20%	18%	**57%**	2%	3%	6%	5%	8%	33%	4%	10%	43%	7%	7%	7%	19%	32%	5%
Gracilibacillus timonensis 03249	7%	47%	29%	47%	48%	48%	**50%**	47%	7%	16%	43%	18%	19%	18%	8%	14%	14%	18%	17%	30%	4%	5%	7%	7%	6%	37%	7%	11%	34%	7%	7%	7%	17%	46%	6%
Halobacillus timonensis 03684	7%	46%	30%	**54%**	**54%**	48%	50%	**54%**	7%	19%	44%	16%	17%	16%	8%	14%	14%	17%	17%	29%	3%	5%	6%	7%	7%	36%	5%	8%	33%	5%	7%	7%	14%	52%	4%
Halophilibacterium massiliense 02839	7%	47%	30%	55%	**56%**	49%	50%	55%	7%	16%	43%	15%	16%	15%	9%	14%	14%	19%	18%	29%	5%	5%	5%	5%	7%	36%	7%	9%	33%	9%	8%	8%	16%	55%	6%
Ihuprevotella massiliensis 00835	9%	15%	16%	15%	15%	15%	16%	15%	6%	25%	15%	35%	35%	35%	7%	**100%**	**99%**	25%	24%	16%	5%	4%	4%	5%	8%	20%	4%	7%	18%	6%	4%	4%	26%	15%	6%
Ihuprevotella massiliensis 00839	9%	15%	16%	15%	15%	15%	16%	15%	6%	25%	15%	35%	35%	35%	7%	**100%**	**99%**	25%	24%	16%	5%	4%	4%	5%	8%	20%	4%	7%	18%	6%	4%	4%	26%	15%	6%
Ihuprevotella massiliensis 00843	9%	15%	16%	15%	15%	15%	16%	15%	6%	25%	15%	35%	35%	35%	6%	**99%**	**99%**	25%	24%	16%	5%	4%	4%	5%	8%	20%	4%	7%	18%	6%	4%	4%	26%	15%	6%
Lentibacillus timonensis 03906	6%	50%	30%	52%	**53%**	49%	51%	52%	6%	18%	43%	16%	17%	16%	9%	17%	17%	18%	18%	29%	3%	4%	5%	6%	7%	40%	7%	9%	34%	7%	8%	8%	18%	52%	5%
Massilibacterium senegalense 00382	7%	50%	33%	**56%**	**56%**	50%	52%	56%	7%	18%	44%	16%	16%	16%	8%	14%	14%	18%	18%	31%	5%	6%	5%	5%	7%	40%	7%	10%	33%	8%	8%	8%	16%	54%	5%
Microvirga massiliensis 00933	7%	35%	44%	35%	35%	34%	34%	35%	5%	22%	31%	22%	23%	22%	5%	21%	21%	22%	20%	42%	6%	4%	5%	5%	7%	42%	6%	9%	**59%**	8%	8%	8%	24%	37%	6%
Microvirga massiliensis 01665	5%	6%	5%	6%	6%	7%	7%	6%	10%	2%	6%	4%	4%	4%	5%	3%	3%	3%	4%	4%	**50%**	**50%**	7%	8%	5%	5%	7%	4%	6%	13%	12%	12%	5%	5%	7%
Numidum massiliensis 03399	6%	59%	31%	50%	51%	70%	**72%**	50%	7%	17%	44%	18%	19%	18%	8%	16%	16%	20%	20%	32%	4%	4%	7%	7%	6%	41%	7%	9%	36%	8%	8%	8%	21%	55%	5%
Oceanibacillus massiliensis 03406	7%	51%	30%	53%	53%	53%	55%	54%	8%	18%	42%	20%	20%	20%	9%	14%	14%	18%	18%	29%	3%	5%	7%	7%	8%	38%	6%	9%	31%	8%	8%	8%	16%	**57%**	6%
Oceanibacillus timonensis 04067	7%	47%	30%	45%	47%	46%	**48%**	45%	7%	16%	43%	17%	17%	16%	7%	13%	13%	17%	18%	30%	3%	4%	6%	7%	6%	36%	6%	11%	34%	8%	5%	5%	17%	45%	5%
Oceanobacillus jeddahense 01891	7%	**53%**	31%	48%	50%	48%	49%	48%	8%	18%	44%	18%	18%	18%	7%	13%	13%	18%	18%	30%	4%	5%	6%	5%	7%	38%	7%	9%	38%	6%	6%	6%	17%	**53%**	6%
Oceanobacillus senegalensis 04097	7%	**68%**	30%	50%	50%	65%	65%	51%	6%	17%	44%	17%	18%	17%	9%	16%	16%	18%	19%	30%	4%	4%	7%	8%	6%	38%	7%	10%	32%	7%	8%	8%	17%	54%	5%
Ottowia massiliensis 01396	4%	6%	6%	4%	5%	5%	5%	4%	9%	5%	5%	7%	7%	6%	7%	5%	5%	4%	5%	3%	**55%**	53%	7%	8%	3%	6%	8%	3%	5%	13%	13%	13%	5%	4%	10%
Paenibacillus cagae 04128	6%	39%	28%	**48%**	46%	40%	41%	**48%**	6%	12%	41%	14%	14%	14%	6%	14%	14%	16%	15%	27%	3%	4%	4%	5%	6%	32%	6%	7%	28%	6%	7%	7%	14%	45%	3%
Parabacteroides timonensis 02340	7%	17%	18%	17%	18%	16%	16%	18%	4%	38%	19%	**50%**	**50%**	**50%**	5%	33%	34%	36%	33%	17%	4%	4%	5%	5%	7%	20%	5%	10%	19%	5%	6%	6%	36%	19%	6%
Prevotella ihuae 00671	9%	15%	16%	14%	15%	15%	16%	15%	6%	25%	15%	35%	35%	35%	7%	**100%**	**100%**	25%	24%	16%	5%	4%	4%	5%	8%	20%	4%	7%	18%	6%	4%	4%	26%	15%	6%
Prevotella lascolaii 02148	9%	15%	16%	15%	15%	15%	16%	15%	6%	25%	15%	35%	35%	35%	7%	**99%**	**99%**	25%	24%	16%	5%	4%	4%	5%	8%	20%	4%	7%	18%	6%	4%	4%	26%	15%	6%
Prevotella merdae 01254	9%	16%	17%	14%	15%	15%	16%	14%	6%	26%	15%	36%	36%	36%	7%	**96%**	**96%**	25%	25%	16%	6%	4%	4%	6%	8%	21%	4%	7%	18%	6%	4%	4%	27%	16%	5%
Pseudomonas massiliensis 01017	8%	37%	36%	34%	33%	35%	36%	34%	5%	21%	30%	22%	22%	21%	8%	18%	18%	18%	20%	37%	6%	6%	6%	6%	6%	**56%**	6%	7%	43%	8%	8%	8%	18%	39%	6%
Rasbobacterium massiliensis 04418	9%	32%	**47%**	33%	32%	32%	32%	33%	6%	18%	28%	19%	19%	18%	7%	16%	16%	20%	21%	34%	5%	6%	5%	6%	8%	42%	5%	8%	43%	8%	8%	8%	21%	34%	4%
Rasbobacterium massiliensis 04419	8%	33%	**47%**	31%	31%	29%	30%	31%	5%	16%	28%	19%	19%	18%	6%	14%	14%	17%	16%	29%	4%	4%	5%	4%	9%	39%	6%	8%	41%	6%	9%	9%	16%	33%	5%
Rasbobacterium massiliensis 04420	8%	28%	**40%**	26%	27%	25%	26%	26%	4%	14%	24%	16%	16%	16%	5%	14%	14%	15%	13%	25%	3%	4%	5%	4%	8%	32%	6%	7%	36%	5%	8%	8%	14%	27%	5%
Rasbobacterium massiliensis 04793	4%	6%	7%	6%	7%	7%	7%	6%	12%	4%	6%	6%	6%	6%	8%	5%	5%	5%	5%	5%	50%	**51%**	9%	9%	4%	8%	7%	3%	7%	14%	13%	13%	6%	7%	8%
Rasbobacterium massiliensis 06881	39%	5%	7%	6%	6%	5%	5%	6%	4%	6%	5%	6%	6%	6%	3%	8%	8%	8%	7%	6%	3%	5%	3%	4%	**57%**	7%	4%	5%	7%	7%	5%	5%	7%	6%	3%
Rubeoparvulum massiliense 00929	8%	49%	30%	**67%**	66%	50%	51%	68%	5%	19%	47%	17%	17%	17%	8%	17%	17%	19%	20%	31%	3%	5%	6%	5%	7%	41%	9%	9%	34%	7%	7%	7%	18%	56%	5%
Sanguibacteroides massiliensis 00542	7%	15%	17%	15%	15%	16%	16%	15%	3%	36%	16%	**54%**	53%	53%	7%	34%	34%	35%	33%	15%	5%	6%	4%	4%	7%	20%	5%	8%	20%	4%	6%	6%	34%	18%	6%
Sediminibacillus massiliensis 01458	7%	49%	30%	53%	**56%**	48%	49%	54%	7%	16%	44%	17%	17%	17%	8%	14%	14%	18%	17%	30%	4%	5%	5%	6%	7%	39%	6%	9%	35%	8%	7%	7%	15%	55%	6%
Tidjanibacter massiliensis 00485	10%	18%	20%	17%	18%	17%	18%	17%	4%	39%	20%	**52%**	**52%**	**52%**	6%	34%	34%	31%	30%	17%	4%	5%	3%	2%	9%	20%	4%	7%	21%	5%	6%	6%	33%	19%	5%
Vaginibacter massiliensis 02368	4%	10%	10%	9%	10%	10%	11%	10%	5%	8%	9%	10%	10%	10%	3%	9%	9%	10%	9%	9%	2%	2%	4%	6%	6%	9%	4%	**72%**	11%	5%	3%	3%	8%	8%	5%
Vaginibacter massiliensis 02382	5%	6%	5%	5%	5%	6%	5%	5%	8%	6%	8%	4%	4%	4%	32%	6%	6%	5%	4%	4%	10%	8%	25%	26%	4%	6%	**47%**	4%	6%	9%	8%	8%	6%	5%	43%
Virgibacillus dakarensis 02401	6%	59%	30%	50%	51%	**70%**	**70%**	51%	6%	15%	45%	16%	16%	16%	6%	15%	15%	17%	17%	30%	5%	5%	6%	7%	7%	39%	5%	8%	34%	8%	8%	8%	18%	55%	4%
Virgibacillus massiliensis 00984	5%	59%	28%	50%	50%	**66%**	**66%**	51%	6%	17%	45%	18%	18%	18%	8%	15%	15%	19%	19%	30%	6%	5%	5%	6%	7%	37%	6%	9%	33%	6%	8%	8%	17%	52%	3%
Vitreoscilla massiliensis 02721	6%	6%	7%	8%	6%	8%	8%	8%	19%	6%	7%	6%	6%	6%	9%	7%	7%	7%	6%	4%	11%	12%	9%	9%	4%	6%	10%	5%	6%	27%	**56%**	**56%**	5%	6%	8%
Xanthomonas massiliensis 02091	8%	7%	6%	7%	7%	9%	8%	7%	17%	4%	4%	6%	6%	6%	7%	5%	5%	4%	6%	5%	14%	14%	9%	9%	7%	6%	10%	4%	7%	**63%**	27%	27%	5%	6%	9%

Colour legend for bacterial names:

Red - Less than 70% identity with best blast hit and are distinct from already known beta-lactamases ARGs within the phylogeny.

Light blue - Greater than or equal to 70% identity with best blast hits

Green - Less than 70% identity with best blast hit and grouped with known ARG according to phylogeny.

Table S3: Distribution of the predicted *fos* genes in the different *fos* gene categories on the basis of pairwise sequences similarity: red for similarity greater than 70%, orange for similarity between 50 and 69%, yellow for similarity between 30 and 49% and green for similarity less than 29%.

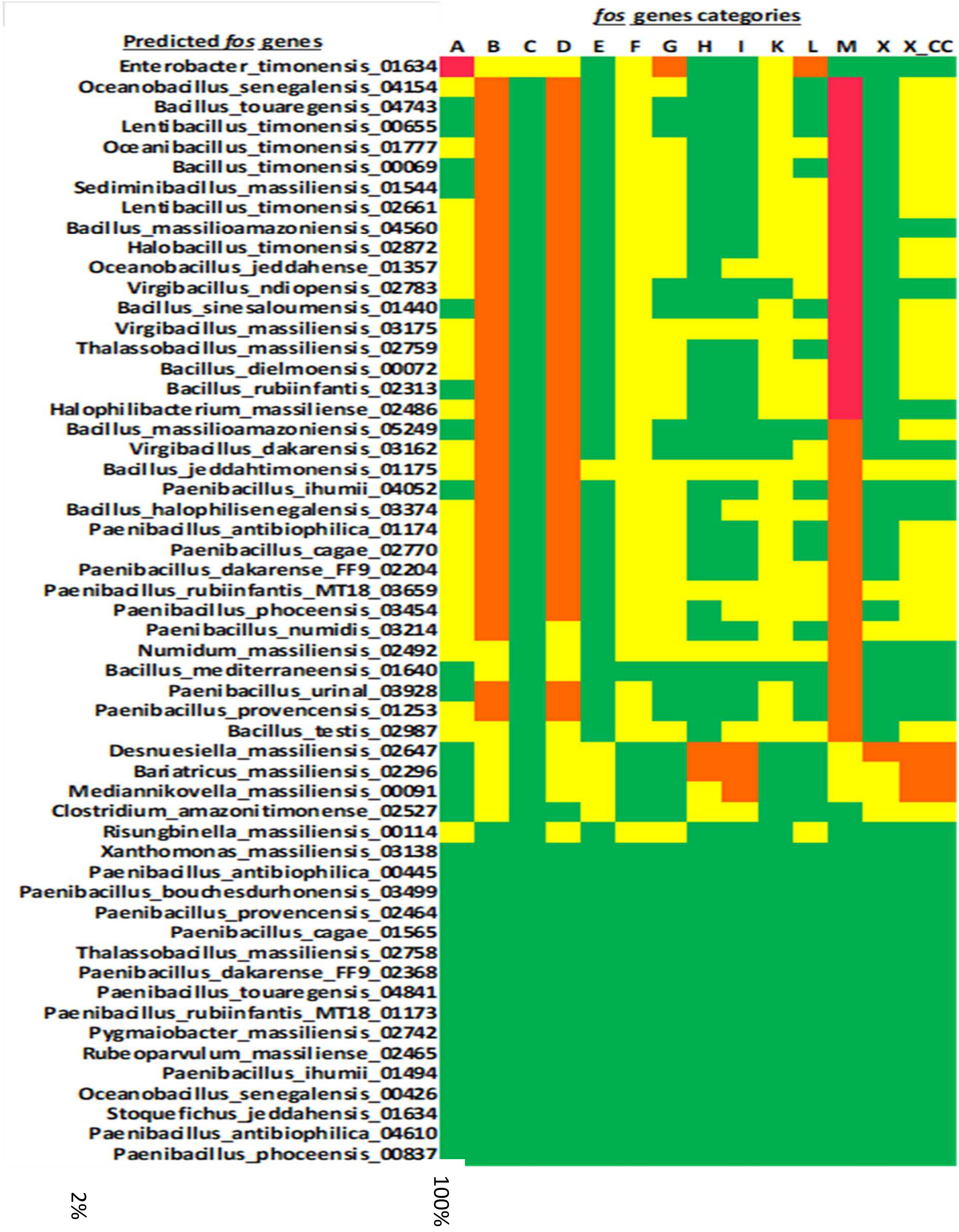

Figure S1: The difference in GC content of ARGs and the host genomes in which they were found.

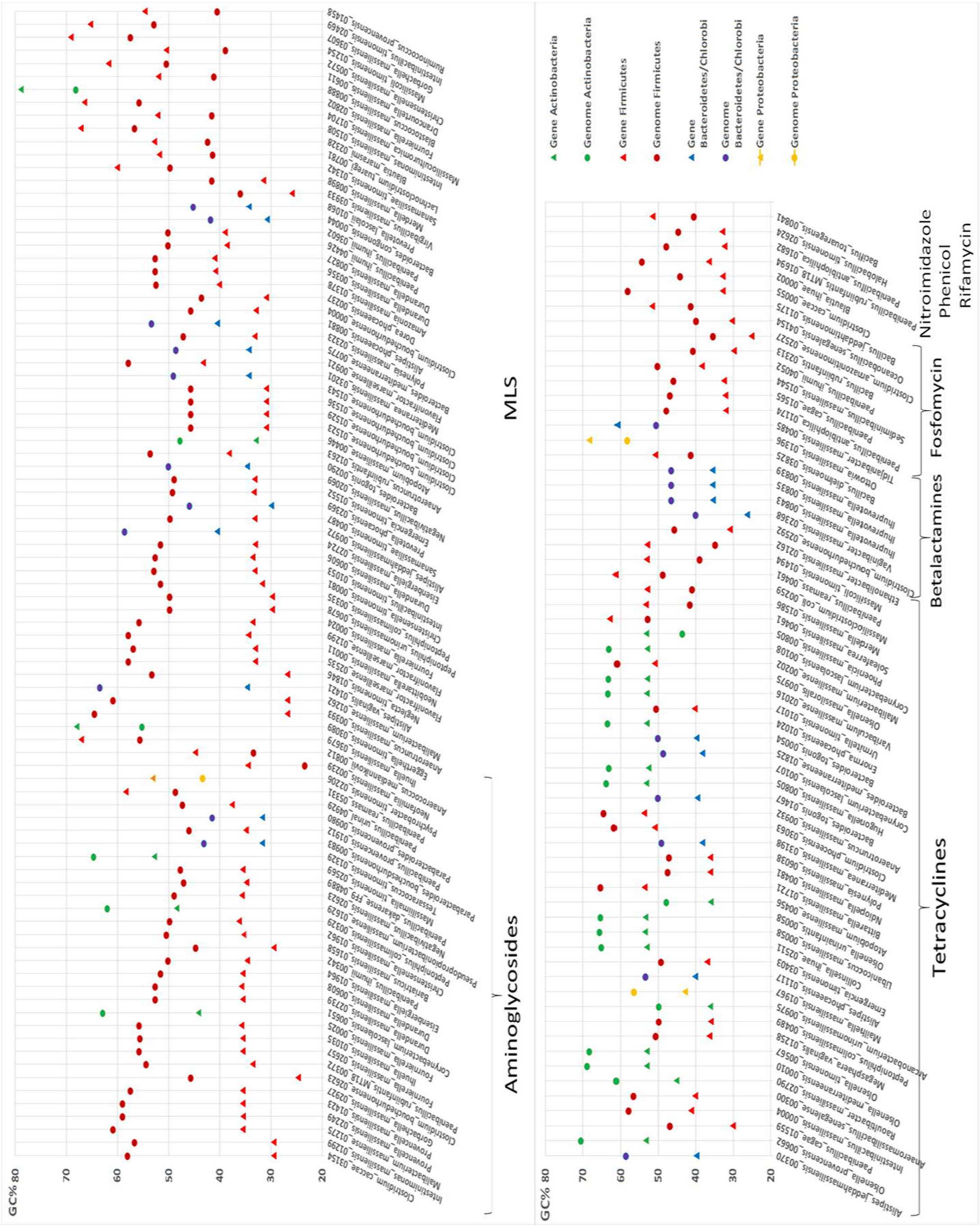

Figure S4

Phylogenetic analysis

Figure S 2-7: Maximum likelihood tree of MLS resistance genes.

Figure S8: Maximum likelihood tree of tetracycline resistance genes.

Figure S9-10: Maximum likelihood tree of Beta-lactamases resistance genes.

Figure S11-14: Maximum likelihood tree of aminoglycosides resistance genes.

Figure S15: Maximum likelihood tree of imidazole resistance genes.

Figure S16-17: Maximum likelihood tree of rifamycin resistance genes.

Figure S18: Maximum likelihood tree of fosfomycin resistance genes.

Figure S19: Maximum likelihood tree of chloramphenicol resistance genes.

Figure S2

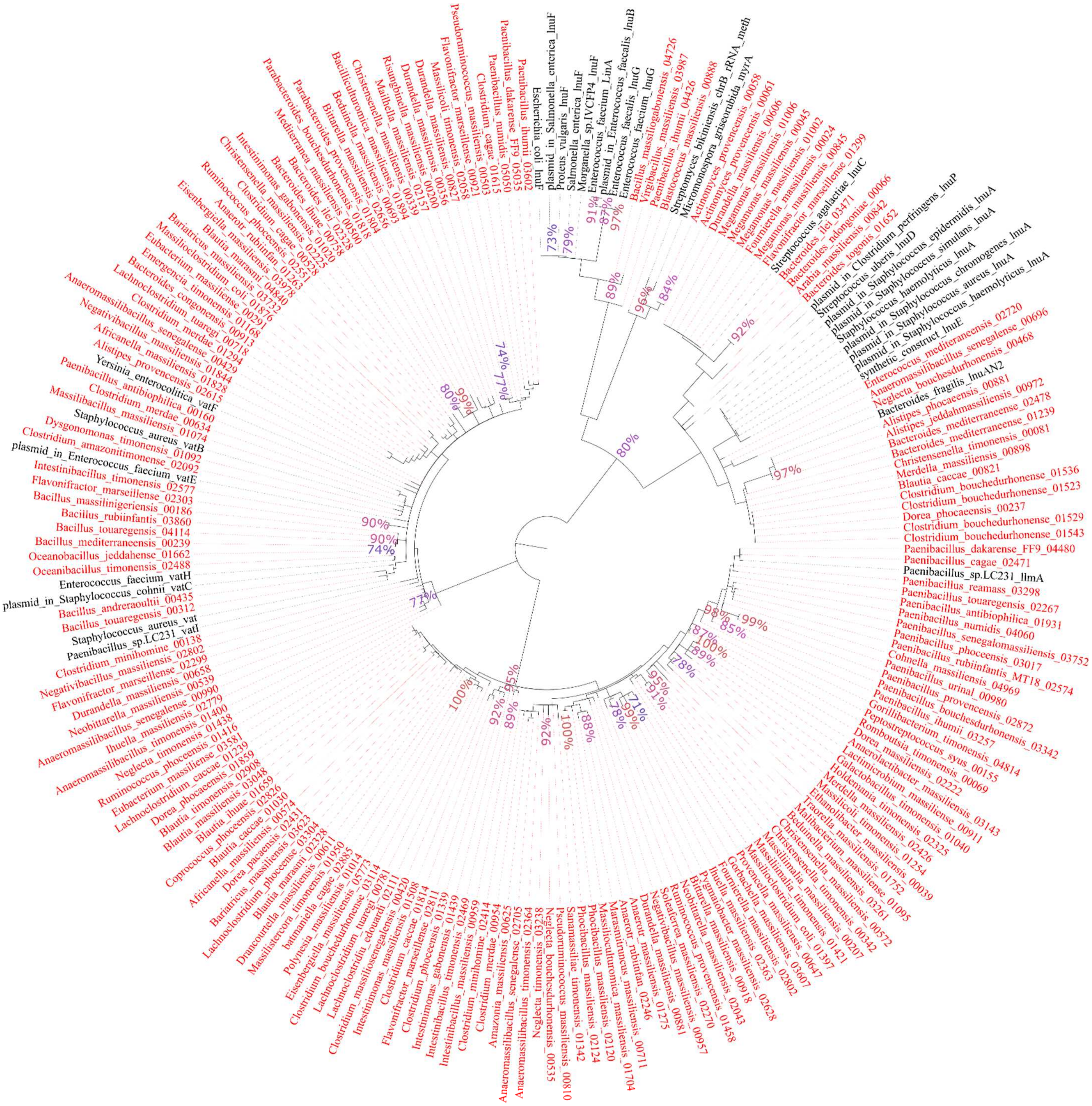
Escherichia_coli_lnuF
plasmid_in_Salmonella_enterica_lnuF
Proteus_vulgaris_lnuF
Salmonella_enterica_lnuF
Morganella_sp.IVCFP4_lnuF
Enterococcus_faecium_LinA
plasmid_in_Enterococcus_faecalis_lnuB
Enterococcus_faecalis_lnuG
Enterococcus_faecium_lnuG
Bacillus_massiliogabonensis_04726
Virgibacillus_massiliensis_03987
Paenibacillus_ihumii_04426
Blastococcus_massiliensis_00888
Streptomyces_bikiniensis_chrB_rRNA_meth
Micromonospora_griseorubida_myrA
Actinomyces_provencensis_00058
Actinomyces_provencensis_00061
Durandella_massiliensis_00606
Megamonas_massiliensis_01006
Megamonas_massiliensis_00045
Megamonas_massiliensis_01002
Fournierella_massiliensis_00024
Megamonas_massiliensis_00845
Flavonifractor_marseillense_01299
Streptococcus_agalactiae_lnuC
Bacteroides_ilei_03471
Bacteroides_ndongoniae_00066
Arabia_massiliensis_00842
Bacteroides_togonis_01652
plasmid_in_Clostridium_perfringens_lnuP
Streptococcus_uberis_lnuD
plasmid_in_Staphylococcus_epidermidis_lnuA
plasmid_in_Staphylococcus_simulans_lnuA
Staphylococcus_haemolyticus_lnuA
plasmid_in_Staphylococcus_chromogenes_lnuA
plasmid_in_Staphylococcus_aureus_lnuA
plasmid_in_Staphylococcus_haemolyticus_lnuA
synthetic_construct_lnuE
Enterococcus_mediteraneensis_02720
Anaeromassilibacillus_senegalense_00696
Neglecta_bouchesdurhonensis_00468
Bacteroides_fragilis_lnuAN2
Alistipes_phocaeensis_00881
Alistipes_jeddahmassiliensis_00972
Bacteroides_mediterraneense_02478
Bacteroides_mediterraneense_01239
Christensenella_timonensis_00081
Merdella_massiliensis_00898
Blautia_caccae_00821
Clostridium_bouchedurhonense_01536
Clostridium_bouchedurhonense_01523
Dorea_phocaeensis_00237
Clostridium_bouchedurhonense_01529
Clostridium_bouchedurhonense_01543
Paenibacillus_dakarense_FF9_04480
Paenibacillus_cagae_02471
Paenibacillus_sp.LC231_llmA
Paenibacillus_reamass_03298
Paenibacillus_touaregensis_02267
Paenibacillus_antibiophilica_01931
Paenibacillus_numidis_04060
Paenibacillus_senegalomassiliensis_03752
Paenibacillus_phoceensis_03017
Paenibacillus_rubiinfantis_MT18_02574
Cohnella_massiliensis_04969
Paenibacillus_urinal_00980
Paenibacillus_provencensis_02872
Paenibacillus_bouchesdurhonensis_03342
Paenibacillus_ihumii_03257
Gorillibacterium_timonensis_04814
Peptostreptococcus_syus_00155
Romboutsia_timonensis_00069
Dorea_massiliensis_02222
Anaerolactibacter_massiliensis_03143
Lactimicrobium_massiliense_00911
Galactobacillus_timonensis_01040
Holdemania_timonensis_02325
Merdella_massiliensis_02426
Massilicoli_timonensis_01254
Ethanolibacter_massiliensis_00039
Traorella_massiliensis_01752
Malibacterium_massiliense_01095
Beduinella_massiliensis_03261
Christensenella_massiliensis_00572
Christensenella_timonensis_00342
Massiliimalia_timonensis_00207
Massilioclostridium_coli_01421
Massiliimalia_massiliensis_01397
Provencella_massiliensis_00647
Gorbachella_massiliensis_03607
Fournierella_massiliensis_02802
Ihuella_massiliensis_02628
Pygmaiobacter_massiliensis_02363
Bittarella_massiliensis_00918
Neobittarella_massiliensis_02043
Ruminococcus_provencensis_01458
Soleaferrea_massiliensis_02270
Negativibacillus_massiliensis_00957
Durandella_massiliensis_00881
Anaerotr_rubiinfan_01275
Anaerotruncus_massiliensis_02246
Marasmitruncus_massiliensis_00711
Massiliculturomica_massiliensis_01704
Phocibacillus_massiliensis_02120
Phocibacillus_massiliensis_02124
Sanamassiliae_timonensis_01342
Pseudoruminococcus_massiliensis_00810
Neglecta_bouchesdurhonensis_00535
Neglecta_timonensis_03238
Anaeromassilibacillus_timonensis_02364
Anaeromassilibacillus_senegalense_02705
Amazonia_massiliensis_00625
Clostridium_merdae_00954
Clostridium_minihomine_02414
Clostridium_massiliensis_00959
Intestinibacillus_timonensis_02469
Intestinimonas_gabonensis_01439
Clostridium_phoceensis_01339
Flavonifractor_marseillense_02811
Clostridium_caccae_01814
Intestinimonas_massiliensis_01508
Clostridium_massiliosenegalensis_00420
Lachnoclostridiu_edouardi_02111
Lachnoclostridium_tuaregi_00781
Clostridium_bouchedurhonense_03114
Eisenbergiella_massiliensis_05773
Polynesia_massiliensis_01014
batmaniella_cagae_02885
Massilistercora_timonensis_01950
Drancourtella_massiliensis_00611
Blautia_marasmi_02328
Lachnoclostridium_phoceense_03304
Bariatricus_massiliensis_03623
Dorea_pacaensis_02431
Africanella_massiliensis_00574
Blautia_caccae_01030
Coprococcus_phoceensis_02826
Blautia_ihuae_01659
Blautia_massiliensis_03048
Blautia_timonensis_02908
Dorea_phocaeensis_01859
Lachnoclostridium_caccae_01239
Eubacterium_massiliense_03581
Ruminococcus_phoceensis_01416
Neglecta_timonensis_01438
Anaeromassilibacillus_timonensis_01400
Ihuella_massiliensis_02779
Anaeromassilibacillus_senegalense_00990
Neobittarella_massiliensis_00539
Durandella_massiliensis_00658
Flavonifractor_marseillense_02299
Negativibacillus_massiliensis_02802
Clostridium_minihomine_00138
Paenibacillus_sp.LC231_vatI
Staphylococcus_aureus_vat
Bacillus_touaregensis_00312
Bacillus_andreraoultii_00435
plasmid_in_Staphylococcus_cohnii_vatC
Enterococcus_faecium_vatH
Oceanibacillus_timonensis_02488
Oceanobacillus_jeddahense_01662
Bacillus_mediterraneensis_00239
Bacillus_touaregensis_04114
Bacillus_rubiinfantis_03860
Bacillus_massilinigeriensis_00186
Flavonifractor_marseillense_02303
Intestinibacillus_timonensis_02577
plasmid_in_Enterococcus_faecium_vatE
Clostridium_amazonitimonense_02092
Dysgonomonas_timonensis_01092
Staphylococcus_aureus_vatB
Massilibacillus_massiliensis_01074
Clostridium_merdae_00634
Paenibacillus_antibiophilica_00160
Yersinia_enterocolitica_vatF
Alistipes_provencensis_02615
Africanella_massiliensis_01828
Negativibacillus_massiliensis_01844
Anaeromassilibacillus_senegalense_00429
Clostridium_merdae_01294
Lachnoclostridium_tuaregi_00718
Bacteroides_congonensis_00913
Emergencia_timonensis_01168
Eubacterium_massiliense_00291
Massilioclostridium_coli_01876
Bariatricus_massiliensis_03732
Blautia_marasmi_04840
Eisenbergiella_massiliensis_03978
Anaerotr_rubiinfan_01263
Ruminococcus_phoceensis_02551
Clostridium_cagae_00528
Christensenella_massiliensis_02225
Intestinimonas_gabonensis_01020
Bacteroides_ihuae_00758
Bacteroides_ilei_02328
Mediterranea_massiliensis_00500
Parabacteroides_bouchesdurhonensis_01818
Parabacteroides_provencensis_01804
Bittarella_massiliensis
Beduinella_massiliensis_02656
Bacilliculturomica_massiliensis_00693
Christensenella_massiliensis_01894
Mailhella_massiliensis_00339
Risungbinella_massiliensis_02157
Durandella_massiliensis_00200
Durandella_massiliensis_00356
Massilicoli_timonensis_00827
Flavonifractor_marseillense_02058
Pseudoruminococcus_massiliensis_00921
Clostridium_massiliensis_00503
Clostridium_cagae_01615
Paenibacillus_numidis_05050
Paenibacillus_dakarense_FF9_05035
Paenibacillus_ihumii_03602

Figure S3

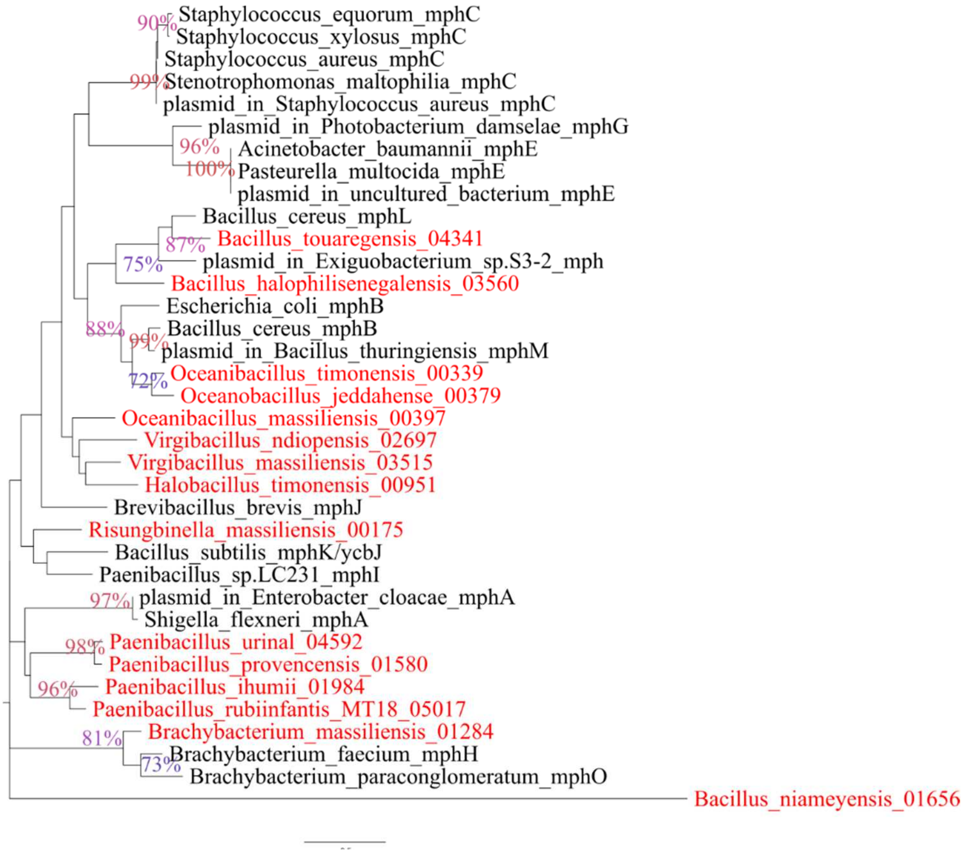

Figure S4

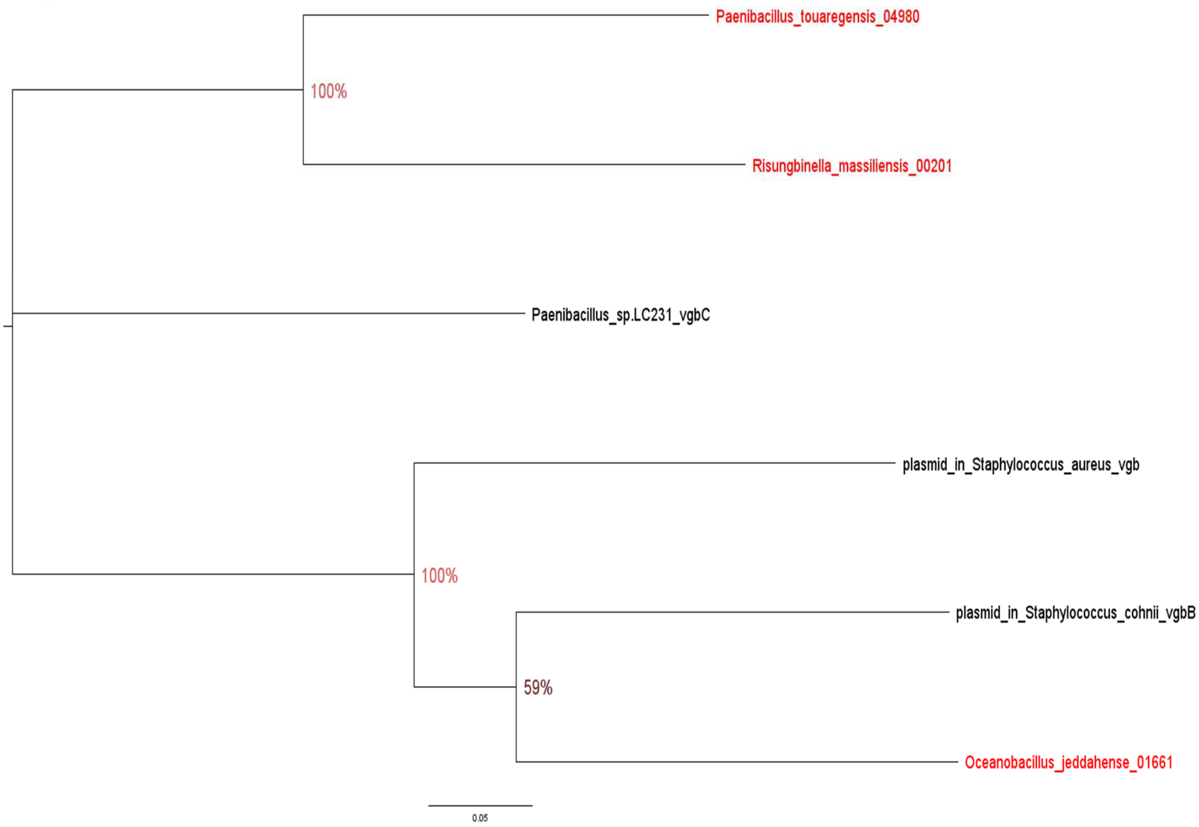

Figure S5

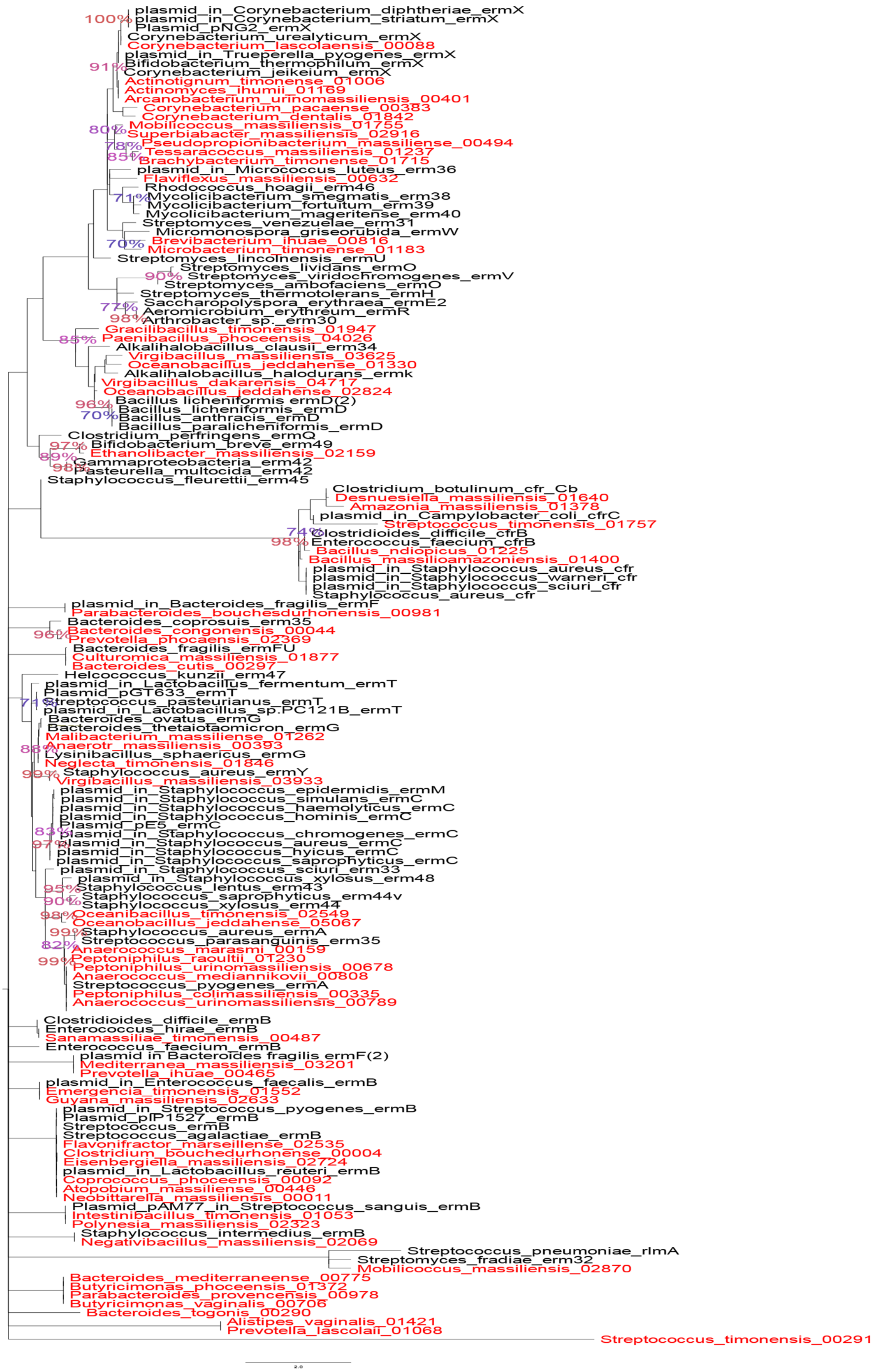
plasmid_in_Corynebacterium_diphtheriae_ermX
plasmid_in_Corynebacterium_striatum_ermX
Plasmid_pNG2_ermX
Corynebacterium_urealyticum_ermX
Corynebacterium_lascolaensis_00088
plasmid_in_Trueperella_pyogenes_ermX
Bifidobacterium_thermophilum_ermX
Corynebacterium_jeikeium_ermX
Actinotignum_timonense_01006
Actinomyces_ihumii_01169
Arcanobacterium_urinomassiliensis_00401
Corynebacterium_pacaense_00383
Corynebacterium_dentalis_01842
Mobilicoccus_massiliensis_01755
Superbiabacter_massiliensis_02916
Pseudopropionibacterium_massiliense_00494
Tessaracoccus_massiliensis_01237
Brachybacterium_timonense_01715
plasmid_in_Micrococcus_luteus_erm36
Flaviflexus_massiliensis_00632
Rhodococcus_hoagii_erm46
Mycolicibacterium_smegmatis_erm38
Mycolicibacterium_fortuitum_erm39
Mycolicibacterium_mageritense_erm40
Streptomyces_venezuelae_erm31
Micromonospora_griseorubida_ermW
Brevibacterium_ihuae_00816
Microbacterium_timonense_01183
Streptomyces_lincolnensis_ermU
Streptomyces_lividans_ermO
Streptomyces_viridochromogenes_ermV
Streptomyces_ambofaciens_ermO
Streptomyces_thermotolerans_ermH
Saccharopolyspora_erythraea_ermE2
Aeromicrobium_erythreum_ermR
Arthrobacter_sp._erm30
Gracilibacillus_timonensis_01947
Paenibacillus_phoceensis_04026
Alkalihalobacillus_clausii_erm34
Virgibacillus_massiliensis_03625
Oceanobacillus_jeddahense_01330
Alkalihalobacillus_halodurans_ermk
Virgibacillus_dakarensis_04717
Oceanobacillus_jeddahense_02824
Bacillus_licheniformis_ermD(2)
Bacillus_licheniformis_ermD
Bacillus_anthracis_ermD
Bacillus_paralicheniformis_ermD
Clostridium_perfringens_ermQ
Bifidobacterium_breve_erm49
Ethanolibacter_massiliensis_02159
Gammaproteobacteria_erm42
Pasteurella_multocida_erm42
Staphylococcus_fleurettii_erm45
Clostridium_botulinum_cfr_Cb
Desnuesiella_massiliensis_01640
Amazonia_massiliensis_01378
plasmid_in_Campylobacter_coli_cfrC
Streptococcus_timonensis_01757
Clostridioides_difficile_cfrB
Enterococcus_faecium_cfrB
Bacillus_ndiopicus_01225
Bacillus_massilioamazoniensis_01400
plasmid_in_Staphylococcus_aureus_cfr
plasmid_in_Staphylococcus_warneri_cfr
plasmid_in_Staphylococcus_sciuri_cfr
Staphylococcus_aureus_cfr
plasmid_in_Bacteroides_fragilis_ermF
Parabacteroides_bouchesdurhonensis_00981
Bacteroides_coprosuis_erm35
Bacteroides_congonensis_00044
Prevotella_phocaensis_02369
Bacteroides_fragilis_ermFU
Culturomica_massiliensis_01877
Bacteroides_cutis_00297
Helcococcus_kunzii_erm47
plasmid_in_Lactobacillus_fermentum_ermT
Plasmid_pGT633_ermT
Streptococcus_pasteurianus_ermT
plasmid_in_Lactobacillus_sp.PC121B_ermT
Bacteroides_ovatus_ermG
Bacteroides_thetaiotaomicron_ermG
Malibacterium_massiliense_01262
Anaerotr_massiliensis_00393
Lysinibacillus_sphaericus_ermG
Neglecta_timonensis_01846
Staphylococcus_aureus_ermY
Virgibacillus_massiliensis_03933
plasmid_in_Staphylococcus_epidermidis_ermM
plasmid_in_Staphylococcus_simulans_ermC
plasmid_in_Staphylococcus_haemolyticus_ermC
plasmid_in_Staphylococcus_hominis_ermC
Plasmid_pE5_ermC
plasmid_in_Staphylococcus_chromogenes_ermC
plasmid_in_Staphylococcus_aureus_ermC
plasmid_in_Staphylococcus_hyicus_ermC
plasmid_in_Staphylococcus_saprophyticus_ermC
plasmid_in_Staphylococcus_sciuri_erm33
plasmid_in_Staphylococcus_xylosus_erm48
Staphylococcus_lentus_erm43
Staphylococcus_saprophyticus_erm44v
Staphylococcus_xylosus_erm44
Oceanibacillus_timonensis_02549
Oceanobacillus_jeddahense_05067
Staphylococcus_aureus_ermA
Streptococcus_parasanguinis_erm35
Anaerococcus_marasmi_00159
Peptoniphilus_raoultii_01230
Peptoniphilus_urinomassiliensis_00678
Anaerococcus_mediannikovii_00808
Streptococcus_pyogenes_ermA
Peptoniphilus_colimassiliensis_00335
Anaerococcus_urinomassiliensis_00789
Clostridioides_difficile_ermB
Enterococcus_hirae_ermB
Sanamassiliae_timonensis_00487
Enterococcus_faecium_ermB
plasmid in Bacteroides fragilis ermF(2)
Mediterranea_massiliensis_03201
Prevotella_ihuae_00465
plasmid_in_Enterococcus_faecalis_ermB
Emergencia_timonensis_01552
Guyana_massiliensis_02633
plasmid_in_Streptococcus_pyogenes_ermB
Plasmid_pIP1527_ermB
Streptococcus_ermB
Streptococcus_agalactiae_ermB
Flavonifractor_marseillense_02535
Clostridium_bouchedurhonense_00004
Eisenbergiella_massiliensis_02724
plasmid_in_Lactobacillus_reuteri_ermB
Coprococcus_phoceensis_00092
Atopobium_massiliense_00446
Neobittarella_massiliensis_00011
Plasmid_pAM77_in_Streptococcus_sanguis_ermB
Intestinibacillus_timonensis_01053
Polynesia_massiliensis_02323
Staphylococcus_intermedius_ermB
Negativibacillus_massiliensis_02069
Streptococcus_pneumoniae_rlmA
Streptomyces_fradiae_erm32
Mobilicoccus_massiliensis_02870
Bacteroides_mediterraneense_00775
Butyricimonas_phoceensis_01372
Parabacteroides_provencensis_00978
Butyricimonas_vaginalis_00706
Bacteroides_togonis_00290
Alistipes_vaginalis_01421
Prevotella_lascolaii_01068
Streptococcus_timonensis_00291
100%
91%
80%
78%
85%
71%
70%
90%
77%
98%
85%
96%
70%
97%
89%
98%
74%
98%
96%
71%
88%
99%
83%
97%
95%
90%
98%
99%
82%
99%
2.0

Figure S6

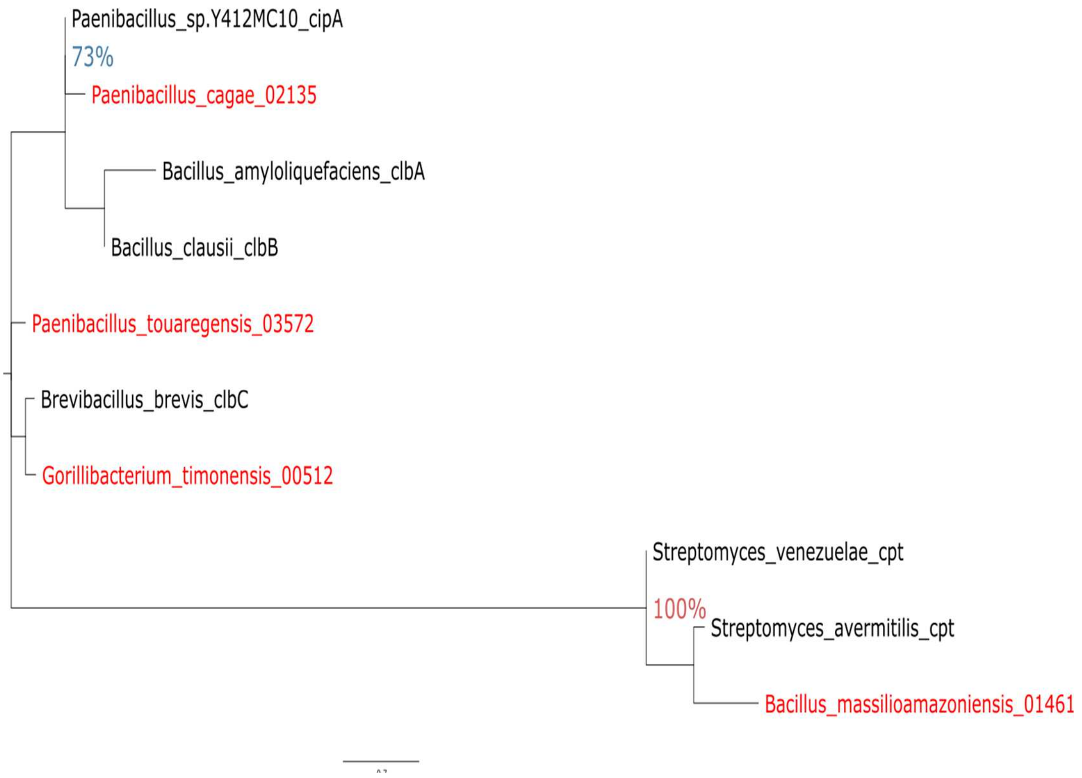

Figure S7

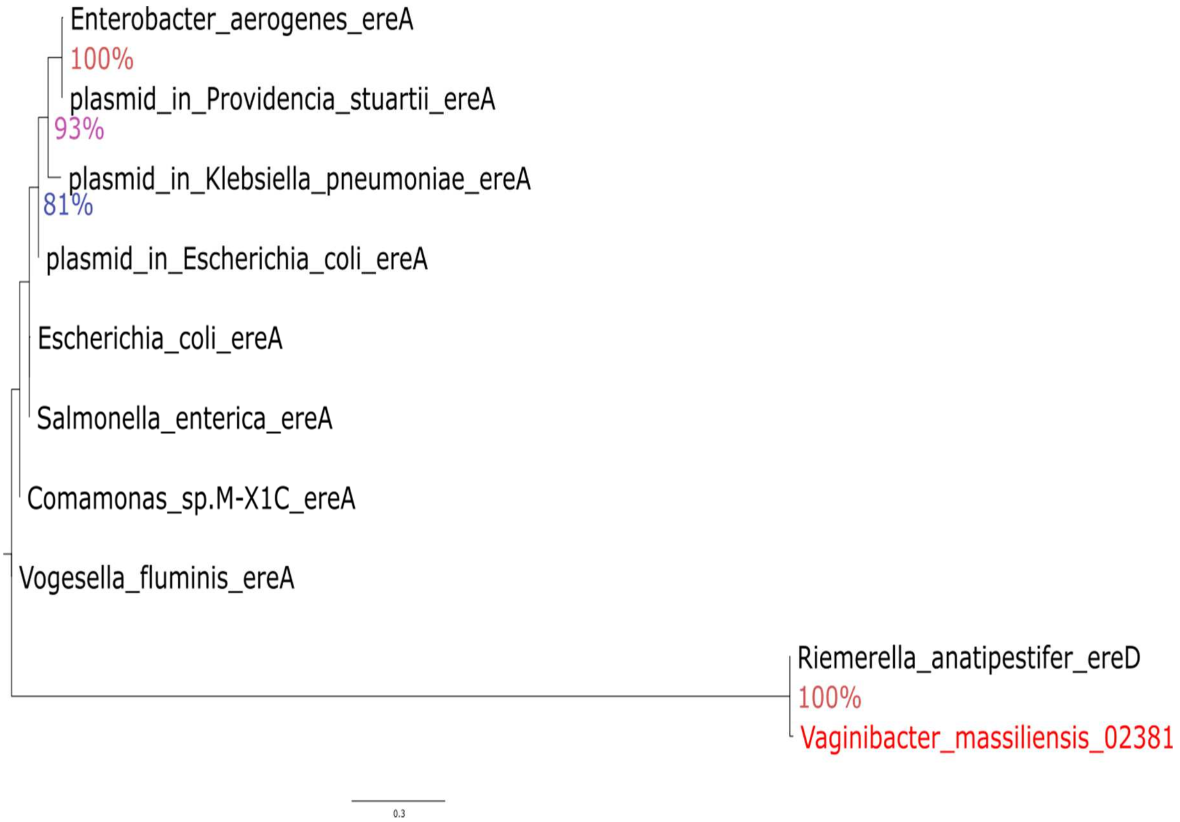

Figure S8

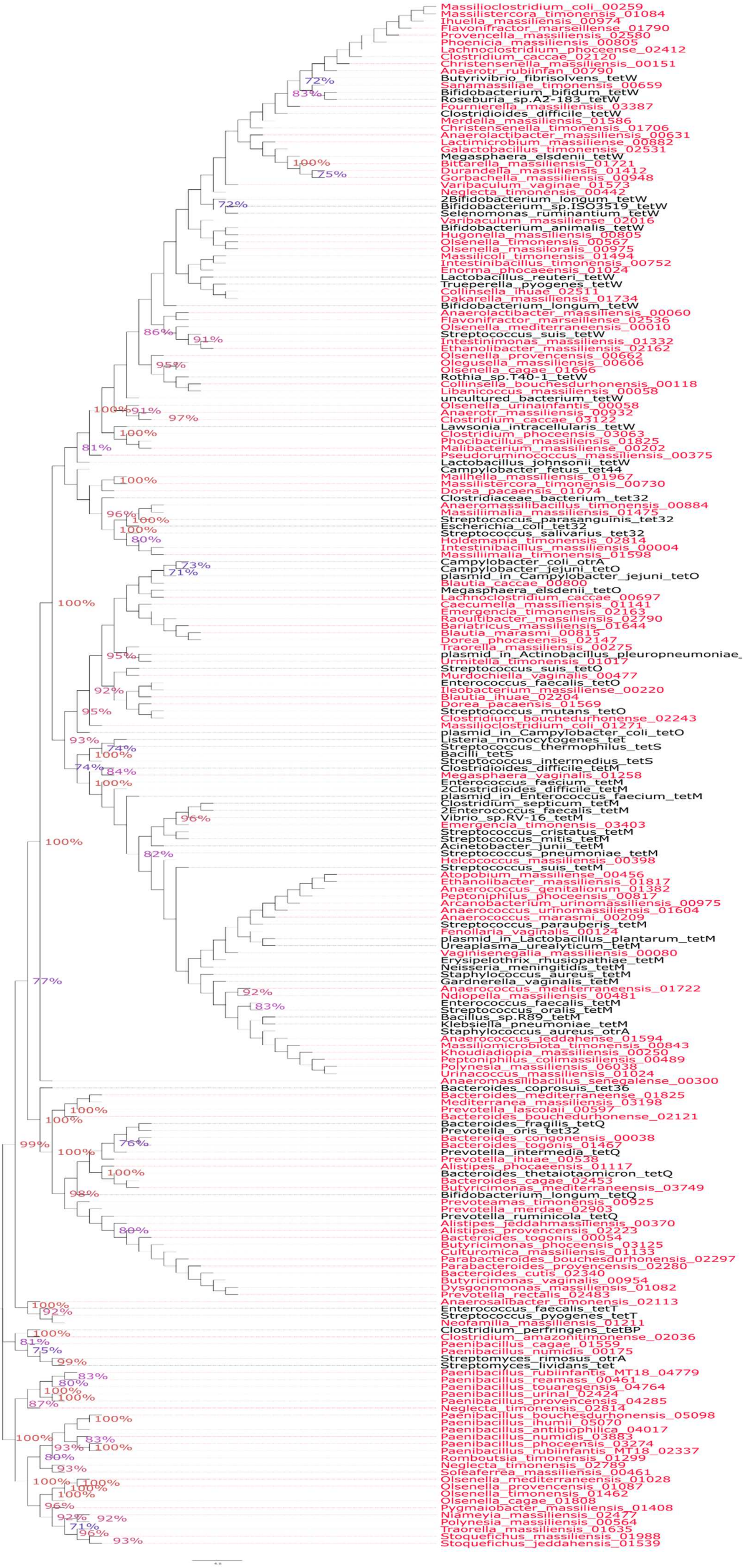
Massilioclostridium_coli_00259
Massilistercora_timonensis_01084
Ihuella_massiliensis_00974
Flavonifractor_marseillense_01790
Provencella_massiliensis_02580
Phoenicia_massiliensis_00805
Lachnoclostridium_phoceense_02412
Clostridium_caccae_02120
Christensenella_massiliensis_00151
Anaerotr_rubiinfan_00790
Butyrivibrio_fibrisolvens_tetW
Sanamassiliae_timonensis_00659
Bifidobacterium_bifidum_tetW
Roseburia_sp.A2-183_tetW
Fournierella_massiliensis_03387
Clostridioides_difficile_tetW
Merdella_massiliensis_01586
Christensenella_timonensis_01706
Anaerolactibacter_massiliensis_00631
Lactimicrobium_massiliense_00882
Galactobacillus_timonensis_02531
Megasphaera_elsdenii_tetW
Bittarella_massiliensis_01721
Durandella_massiliensis_01412
Gorbachella_massiliensis_00948
Varibaculum_vaginae_01573
Neglecta_timonensis_00442
2Bifidobacterium_longum_tetW
Bifidobacterium_sp.ISO3519_tetW
Selenomonas_ruminantium_tetW
Varibaculum_massiliense_02016
Bifidobacterium_animalis_tetW
Hugonella_massiliensis_00805
Olsenella_timonensis_00567
Olsenella_massiloralis_00975
Massilicoli_timonensis_01494
Intestinibacillus_timonensis_00752
Enorma_phocaeensis_01024
Lactobacillus_reuteri_tetW
Trueperella_pyogenes_tetW
Collinsella_ihuae_02511
Dakarella_massiliensis_01734
Bifidobacterium_longum_tetW
Anaerolactibacter_massiliensis_00060
Flavonifractor_marseillense_02536
Olsenella_mediterraneensis_00010
Streptococcus_suis_tetW
Intestinimonas_massiliensis_01332
Ethanolibacter_massiliensis_02162
Olsenella_provencensis_00662
Olegusella_massiliensis_00606
Olsenella_cagae_01666
Rothia_sp.T40-1_tetW
Collinsella_bouchesdurhonensis_00118
Libanicoccus_massiliensis_00058
uncultured_bacterium_tetW
Olsenella_urininfantis_00058
Anaerotr_massiliensis_00932
Clostridium_caccae_03122
Lawsonia_intracellularis_tetW
Clostridium_phoceensis_03063
Phocibacillus_massiliensis_01825
Malibacterium_massiliense_00202
Pseudoruminococcus_massiliensis_00375
Lactobacillus_johnsonii_tetW
Campylobacter_fetus_tet44
Mailhella_massiliensis_01967
Massilistercora_timonensis_00730
Dorea_pacaensis_01074
Clostridiaceae_bacterium_tet32
Anaeromassilibacillus_timonensis_00884
Massilimalia_massiliensis_01475
Streptococcus_parasanguinis_tet32
Escherichia_coli_tet32
Streptococcus_salivarius_tet32
Holdemania_timonensis_02814
Intestinibacillus_massiliensis_00004
Massilimalia_timonensis_01598
Campylobacter_coli_otrA
Campylobacter_jejuni_tetO
plasmid_in_Campylobacter_jejuni_tetO
Blautia_caccae_00800
Megasphaera_elsdenii_tetO
Lachnoclostridium_caccae_00697
Caecumella_massiliensis_01141
Emergencia_timonensis_02163
Raoultibacter_massiliensis_02790
Bariatricus_massiliensis_01644
Blautia_marasmi_00815
Dorea_phocaeensis_02147
Traorella_massiliensis_00275
plasmid_in_Actinobacillus_pleuropneumoniae_
Urmitella_timonensis_01017
Streptococcus_suis_tetO
Murdochiella_vaginalis_00477
Enterococcus_faecalis_tetO
Ileobacterium_massiliense_00220
Blautia_ihuae_02204
Dorea_pacaensis_01569
Streptococcus_mutans_tetO
Clostridium_bouchedurhonense_02243
Massilioclostridium_coli_01271
plasmid_in_Campylobacter_coli_tetO
Listeria_monocytogenes_tet
Streptococcus_thermophilus_tetS
Bacilli_tetS
Streptococcus_intermedius_tetS
Clostridioides_difficile_tetM
Megasphaera_vaginalis_01258
Enterococcus_faecium_tetM
2Clostridioides_difficile_tetM
plasmid_in_Enterococcus_faecium_tetM
Clostridium_septicum_tetM
2Enterococcus_faecalis_tetM
Vibrio_sp.RV-16_tetM
Emergencia_timonensis_03403
Streptococcus_cristatus_tetM
Streptococcus_mitis_tetM
Acinetobacter_junii_tetM
Streptococcus_pneumoniae_tetM
Helcococcus_massiliensis_00398
Streptococcus_suis_tetM
Atopobium_massiliense_00456
Ethanolibacter_massiliensis_01817
Anaerococcus_genitaliorum_01382
Peptoniphilus_phoceensis_00817
Arcanobacterium_urinomassiliensis_00975
Anaerococcus_urinomassiliensis_01604
Anaerococcus_marasmi_00209
Streptococcus_parauberis_tetM
Fenollaria_vaginalis_00124
plasmid_in_Lactobacillus_plantarum_tetM
Ureaplasma_urealyticum_tetM
Vaginisenegalia_massiliensis_00080
Erysipelothrix_rhusiopathiae_tetM
Neisseria_meningitidis_tetM
Staphylococcus_aureus_tetM
Gardnerella_vaginalis_tetM
Anaerococcus_mediterraneensis_01722
Ndiopella_massiliensis_00481
Enterococcus_faecalis_tetM
Streptococcus_oralis_tetM
Bacillus_sp.R89_tetM
Klebsiella_pneumoniae_tetM
Staphylococcus_aureus_otrA
Anaerococcus_jeddahense_01594
Massiliomicrobiota_timonensis_00843
Khoudiadiopia_massiliensis_00250
Peptoniphilus_colimassiliensis_00489
Polynesia_massiliensis_06038
Urinacoccus_massiliensis_01024
Anaeromassilibacillus_senegalense_00300
Bacteroides_coprosuis_tet36
Bacteroides_mediterraneense_01825
Mediterranea_massiliensis_03198
Prevotella_lascolaii_00597
Bacteroides_bouchedurhonense_02121
Bacteroides_fragilis_tetQ
Prevotella_oris_tet32
Bacteroides_congonensis_00038
Bacteroides_togonis_01467
Prevotella_intermedia_tetQ
Prevotella_ihuae_00538
Alistipes_phocaeensis_01117
Bacteroides_thetaiotaomicron_tetQ
Bacteroides_cagae_02453
Butyricimonas_mediterraneensis_03749
Bifidobacterium_longum_tetQ
Prevoteamas_timonensis_00925
Prevotella_merdae_02903
Prevotella_ruminicola_tetQ
Alistipes_jeddahmassiliensis_00370
Alistipes_provencensis_02223
Bacteroides_togonis_00054
Butyricimonas_phoceensis_03125
Culturomica_massiliensis_01133
Parabacteroides_bouchesdurhonensis_02297
Parabacteroides_provencensis_02280
Bacteroides_cutis_02340
Butyricimonas_vaginalis_00954
Dysgonomonas_massiliensis_01082
Prevotella_rectalis_02483
Anaerosalibacter_timonensis_02113
Enterococcus_faecalis_tetT
Streptococcus_pyogenes_tetT
Neofamilia_massiliensis_01211
Clostridium_perfringens_tetBP
Clostridium_amazonitimonense_02036
Paenibacillus_cagae_01559
Paenibacillus_numidis_00175
Streptomyces_rimosus_otrA
Streptomyces_lividans_tet
Paenibacillus_rubiinfantis_MT18_04779
Paenibacillus_reamass_00461
Paenibacillus_touaregensis_04764
Paenibacillus_urinal_02424
Paenibacillus_provencensis_04285
Neglecta_timonensis_02814
Paenibacillus_bouchesdurhonensis_05098
Paenibacillus_ihumii_05070
Paenibacillus_antibiophilica_04017
Paenibacillus_numidis_03883
Paenibacillus_phoceensis_03274
Paenibacillus_rubiinfantis_MT18_02337
Romboutsia_timonensis_01299
Neglecta_timonensis_02789
Soleaferrea_massiliensis_00461
Olsenella_mediterraneensis_01028
Olsenella_provencensis_01087
Olsenella_timonensis_01462
Olsenella_cagae_01808
Pygmaiobacter_massiliensis_01408
Niameyia_massiliensis_02477
Polynesia_massiliensis_00564
Traorella_massiliensis_01635
Stoquefichus_massiliensis_01988
Stoquefichus_jeddahensis_01539

Figure S9

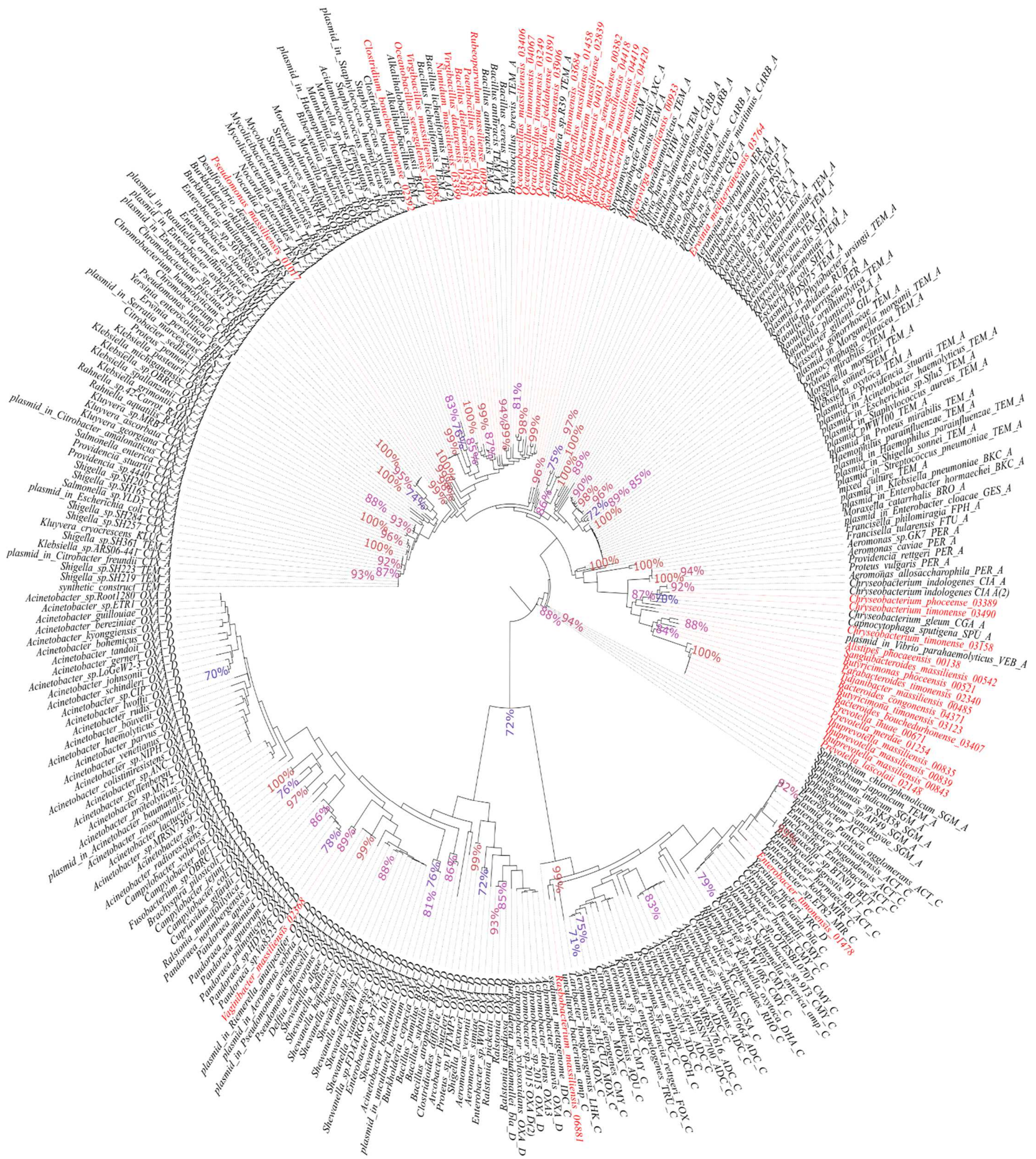

Figure S10

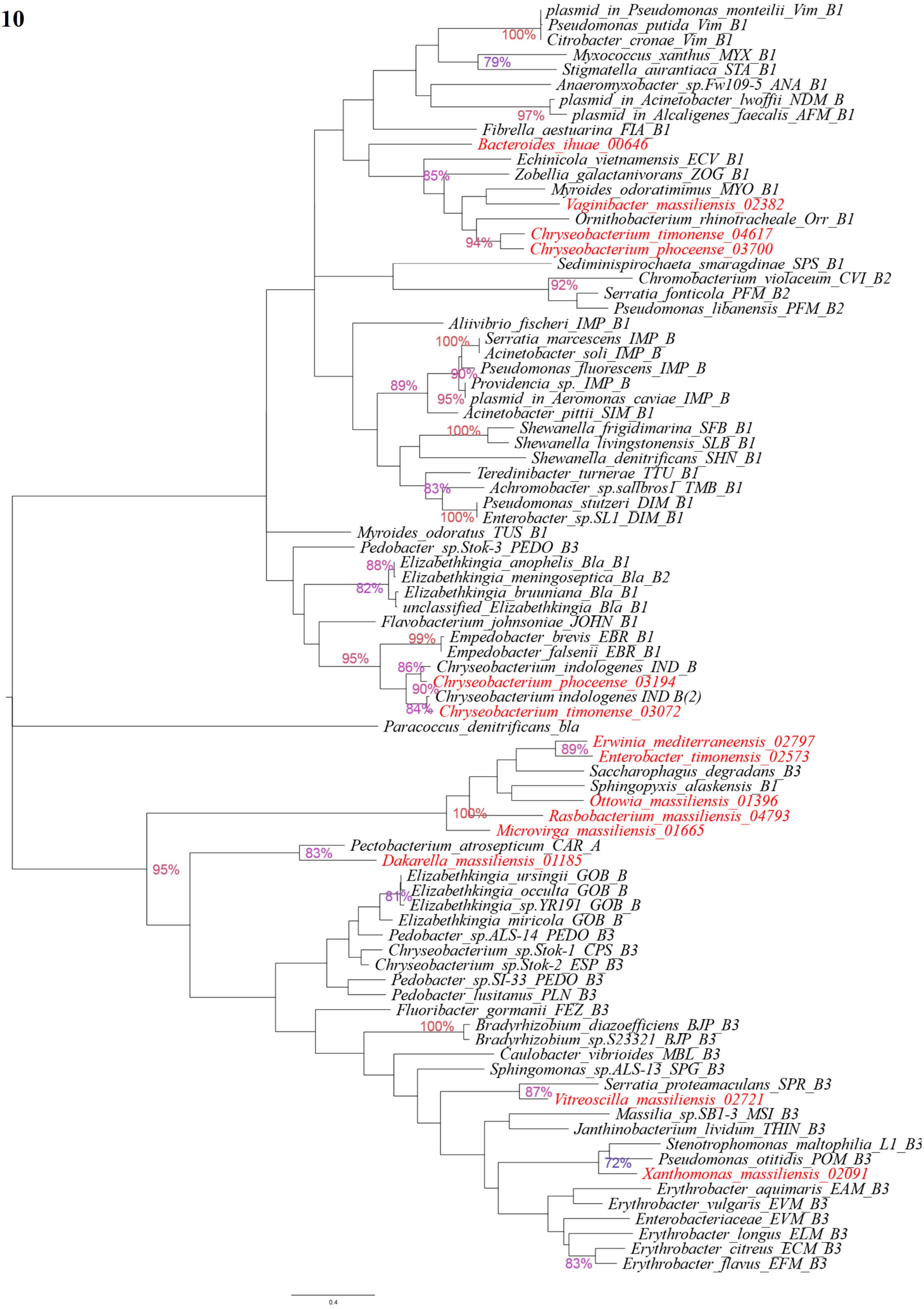

plasmid_in_Pseudomonas_monteilii_Vim_B1
Pseudomonas_putida_Vim_B1
100%
Citrobacter_cronae_Vim_B1
Myxococcus_xanthus_MYX_B1
79%
Stigmatella_aurantiaca_STA_B1
Anaeromyxobacter_sp.Fw109-5_ANA_B1
plasmid_in_Acinetobacter_lwoffii_NDM_B
97%
plasmid_in_Alcaligenes_faecalis_AFM_B1
Fibrella_aestuarina_FIA_B1
Bacteroides_ihuae_00646
Echinicola_vietnamensis_ECV_B1
85%
Zobellia_galactanivorans_ZOG_B1
Myroides_odoratimimus_MYO_B1
Vaginibacter_massiliensis_02382
Ornithobacterium_rhinotracheale_Orr_B1
Chryseobacterium_timonense_04617
94%
Chryseobacterium_phoceense_03700
Sediminispirochaeta_smaragdinae_SPS_B1
Chromobacterium_violaceum_CVI_B2
92%
Serratia_fonticola_PFM_B2
Pseudomonas_libanensis_PFM_B2
Aliivibrio_fischeri_IMP_B1
100%
Serratia_marcescens_IMP_B
Acinetobacter_soli_IMP_B
Pseudomonas_fluorescens_IMP_B
90%
89%
Providencia_sp._IMP_B
95%
plasmid_in_Aeromonas_caviae_IMP_B
Acinetobacter_pittii_SIM_B1
100%
Shewanella_frigidimarina_SFB_B1
Shewanella_livingstonensis_SLB_B1
Shewanella_denitrificans_SHN_B1
Teredinibacter_turnerae_TTU_B1
83%
Achromobacter_sp.sallbros1_TMB_B1
Pseudomonas_stutzeri_DIM_B1
100%
Enterobacter_sp.SL1_DIM_B1
Myroides_odoratus_TUS_B1
Pedobacter_sp.Stok-3_PEDO_B3
88%
Elizabethkingia_anophelis_Bla_B1
Elizabethkingia_meningoseptica_Bla_B2
82%
Elizabethkingia_bruuniana_Bla_B1
unclassified_Elizabethkingia_Bla_B1
Flavobacterium_johnsoniae_JOHN_B1
99%
Empedobacter_brevis_EBR_B1
Empedobacter_falsenii_EBR_B1
95%
86%
Chryseobacterium_indologenes_IND_B
90%
Chryseobacterium_phoceense_03194
Chryseobacterium indologenes IND B(2)
84%
Chryseobacterium_timonense_03072
Paracoccus_denitrificans_bla
89%
Erwinia_mediterraneensis_02797
Enterobacter_timonensis_02573
Saccharophagus_degradans_B3
Sphingopyxis_alaskensis_B1
Ottowia_massiliensis_01396
100%
Rasbobacterium_massiliensis_04793
Microvirga_massiliensis_01665
83%
Pectobacterium_atrosepticum_CAR_A
95%
Dakarella_massiliensis_01185
Elizabethkingia_ursingii_GOB_B
81%
Elizabethkingia_occulta_GOB_B
Elizabethkingia_sp.YR191_GOB_B
Elizabethkingia_miricola_GOB_B
Pedobacter_sp.ALS-14_PEDO_B3
Chryseobacterium_sp.Stok-1_CPS_B3
Chryseobacterium_sp.Stok-2_ESP_B3
Pedobacter_sp.SI-33_PEDO_B3
Pedobacter_lusitanus_PLN_B3
Fluoribacter_gormanii_FEZ_B3
100%
Bradyrhizobium_diazoefficiens_BJP_B3
Bradyrhizobium_sp.S23321_BJP_B3
Caulobacter_vibrioides_MBL_B3
Sphingomonas_sp.ALS-13_SPG_B3
Serratia_proteamaculans_SPR_B3
87%
Vitreoscilla_massiliensis_02721
Massilia_sp.SB1-3_MSI_B3
Janthinobacterium_lividum_THIN_B3
Stenotrophomonas_maltophilia_L1_B3
72%
Pseudomonas_otitidis_POM_B3
Xanthomonas_massiliensis_02091
Erythrobacter_aquimaris_EAM_B3
Erythrobacter_vulgaris_EVM_B3
Enterobacteriaceae_EVM_B3
Erythrobacter_longus_ELM_B3
Erythrobacter_citreus_ECM_B3
83%
Erythrobacter_flavus_EFM_B3
0.4

Figure S11

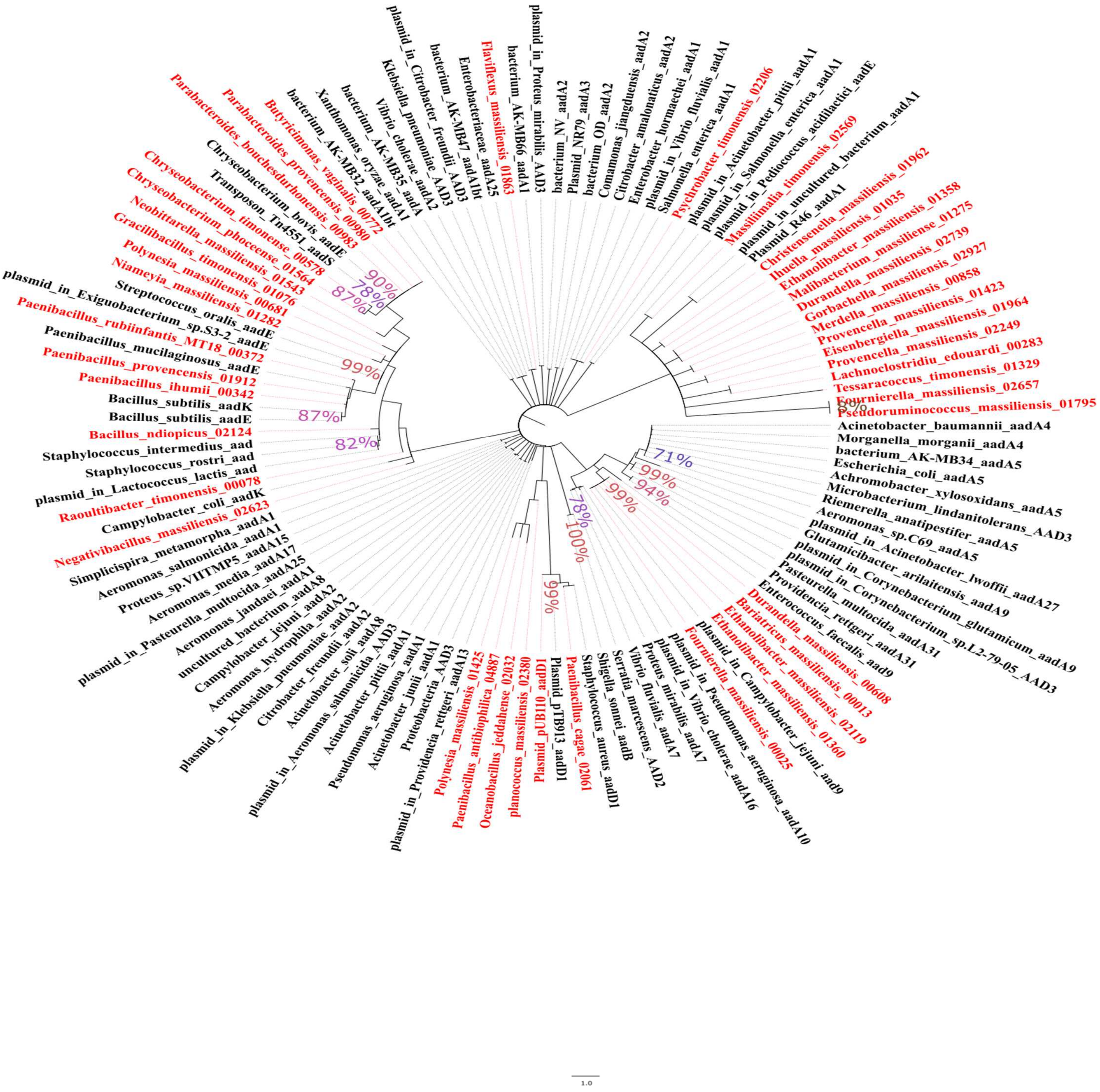

Parabacteroides_bouchesdurhonensis_00983
Parabacteroides_provencensis_00980
Butyricimonas_vaginalis_00772
bacterium_AK-MB32_aadA1bt
Xanthomonas_oryzae_aadA1
bacterium_AK-MB35_aadA
Vibrio_cholerae_aadA2
Klebsiella_pneumoniae_AAD3
plasmid_in_Citrobacter_freundii_AAD3
bacterium_AK-MB47_aadA1bt
Enterobacteriaceae_aadA25
Flaviflexus_massiliensis_01863
bacterium_AK-MB66_aadA1
plasmid_in_Proteus_mirabilis_AAD3
bacterium_NV_aadA2
Plasmid_NR79_aadA3
bacterium_OD_aadA2
Comamonas_jiangduensis_aadA2
Citrobacter_amalonaticus_aadA2
Enterobacter_hormaechei_aadA1
plasmid_in_Vibrio_fluvialis_aadA1
Salmonella_enterica_aadA1
Psychrobacter_timonensis_02206
plasmid_in_Acinetobacter_pittii_aadA1
plasmid_in_Salmonella_enterica_aadA1
plasmid_in_Pediococcus_acidilactici_aadE
Massilimalia_timonensis_02569
plasmid_in_uncultured_bacterium_aadA1
Plasmid_R46_aadA1
Christensenella_massiliensis_01962
Ihuella_massiliensis_01035
Ethanolibacter_massiliensis_01358
Malibacterium_massiliense_01275
Durandella_massiliensis_02739
Gorbachella_massiliensis_02927
Merdella_massiliensis_00858
Provencella_massiliensis_01423
Eisenbergiella_massiliensis_01964
Provencella_massiliensis_02249
Lachnoclostridiu_edouardi_00283
Tessaracoccus_timonensis_01329
Fournierella_massiliensis_02657
Pseudoruminococcus_massiliensis_01795
Acinetobacter_baumannii_aadA4
Morganella_morganii_aadA4
bacterium_AK-MB34_aadA5
Escherichia_coli_aadA5
Achromobacter_xylosoxidans_aadA5
Microbacterium_lindanitolerans_AAD3
Riemerella_anatipestifer_aadA5
Aeromonas_sp.C69_aadA5
plasmid_in_Acinetobacter_lwoffii_aadA27
Glutamicibacter_arilaitensis_aadA9
plasmid_in_Corynebacterium_glutamicum_aadA9
plasmid_in_Corynebacterium_sp.L2-79-05_AAD3
Pasteurella_multocida_aadA31
Providencia_rettgeri_aadA31
Enterococcus_faecalis_aad9
Durandella_massiliensis_00608
Bariatricus_massiliensis_00013
Ethanolibacter_massiliensis_02119
Ethanolibacter_massiliensis_01360
Fournierella_massiliensis_00025
plasmid_in_Campylobacter_jejuni_aad9
plasmid_in_Pseudomonas_aeruginosa_aadA10
plasmid_in_Vibrio_cholerae_aadA16
Proteus_mirabilis_aadA7
Vibrio_fluvialis_aadA7
Serratia_marcescens_AAD2
Shigella_sonnei_aadB
Staphylococcus_aureus_aadD1
Paenibacillus_cagae_02061
Plasmid_pTB913_aadD1
Plasmid_pUB110_aadD1
planococcus_massiliensis_02380
Oceanobacillus_jeddahense_02032
Paenibacillus_antibiophilica_04887
Polynesia_massiliensis_01425
plasmid_in_Providencia_rettgeri_aadA13
Proteobacteria_rettgeri_AAD3
Acinetobacter_junii_aadA1
Pseudomonas_aeruginosa_aadA1
Acinetobacter_pittii_aadA1
plasmid_in_Aeromonas_salmonicida_AAD3
Acinetobacter_soli_aadA8
Citrobacter_freundii_aadA2
plasmid_in_Klebsiella_pneumoniae_aadA2
Aeromonas_hydrophila_aadA2
Campylobacter_jejuni_aadA2
uncultured_bacterium_aadA8
Aeromonas_jandaei_aadA1
plasmid_in_Pasteurella_multocida_aadA25
Aeromonas_media_aadA17
Proteus_sp.VIITMP5_aadA15
Aeromonas_salmonicida_aadA1
Simplicispira_metamorpha_aadA1
Negativibacillus_massiliensis_02623
Campylobacter_coli_aadK
Raoultibacter_timonensis_00078
plasmid_in_Lactococcus_lactis_aad
Staphylococcus_rostri_aad
Staphylococcus_intermedius_aad
Bacillus_ndiopicus_02124
Bacillus_subtilis_aadE
Bacillus_subtilis_aadK
Paenibacillus_ihumii_00342
Paenibacillus_provencensis_01912
Paenibacillus_mucilaginosus_aadE
Paenibacillus_rubiinfantis_MT18_00372
plasmid_in_Exiguobacterium_sp.S3-2_aadE
Streptococcus_oralis_aadE
Niameyia_massiliensis_01282
Polynesia_massiliensis_00681
Gracilibacillus_timonensis_01076
Neobittarella_massiliensis_01543
Chryseobacterium_phocaeense_01564
Chryseobacterium_timonense_00578
Transposon_Tn4551_aadS
Chryseobacterium_bovis_aadE
90%
78%
87%
99%
87%
82%
8%
71%
99%
94%
99%
78%
100%
99%
1.0

Figure S12

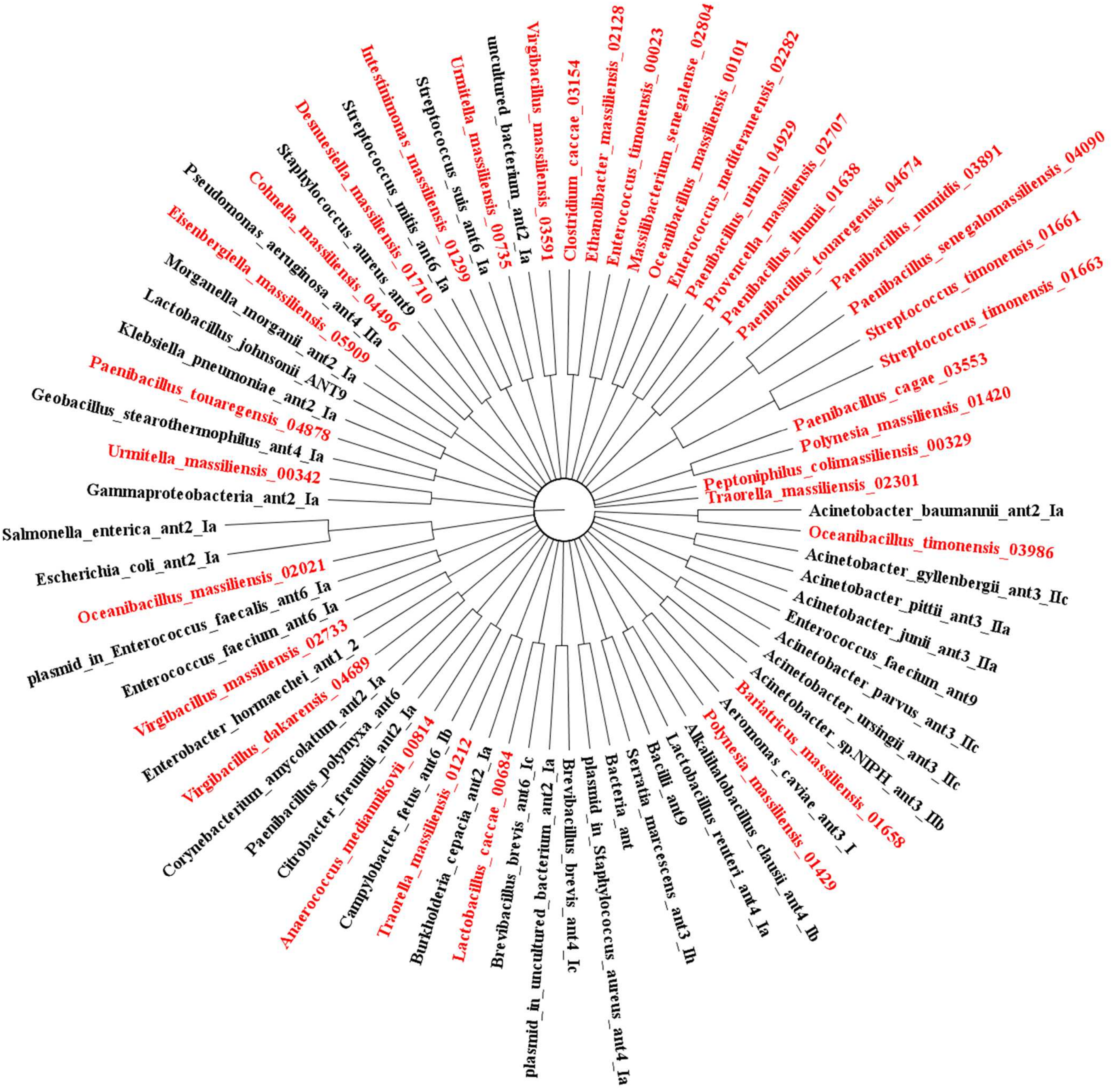

Salmonella_enterica_ant2_Ia
Gammaproteobacteria_ant2_Ia
Urmitella_massiliensis_00342
Geobacillus_stearothermophilus_ant4_Ia
Paenibacillus_touaregensis_04878
Klebsiella_pneumoniae_ant2_Ia
Lactobacillus_johnsonii_ANT9
Morganella_morganii_ant2_Ia
Eisenbergiella_massiliensis_05909
Pseudomonas_aeruginosa_ant4_IIa
Cohnella_massiliensis_04496
Staphylococcus_aureus_ant9
Desnuesiella_massiliensis_01710
Streptococcus_mitis_ant6_Ia
Intestinimonas_massiliensis_01299
Streptococcus_suis_ant6_Ia
Urmitella_massiliensis_00735
uncultured_bacterium_ant2_Ia
Virgibacillus_massiliensis_03591
Clostridium_caccae_03154
Ethanoligenens_massiliensis_02128
Enterococcus_timonensis_00023
Massilibacterium_senegalense_02804
Oceanibacillus_massiliensis_00101
Enterococcus_mediterraneensis_02282
Oceanibacillus_urinal_04929
Provencella_massiliensis_02707
Paenibacillus_ihumii_01638
Paenibacillus_touaregensis_04674
Paenibacillus_numidis_03891
Paenibacillus_senegalomassiliensis_04090
Streptococcus_timonensis_01661
Streptococcus_timonensis_01663
Paenibacillus_cagae_03553
Polynesia_massiliensis_01420
Peptoniphilus_colimassiliensis_00329
Peptoniphilus_
Traorella_massiliensis_02301
Acinetobacter_baumannii_ant2_Ia
Oceanibacillus_timonensis_03986
Acinetobacter_gyllenbergii_ant3_IIc
Acinetobacter_pittii_ant3_IIa
Acinetobacter_junii_ant3_IIa
Enterococcus_faecium_ant9
Acinetobacter_parvus_ant3_IIc
Acinetobacter_ursingii_ant3_IIc
Acinetobacter_sp.NIPH_ant3_IIb
Bariatricus_massiliensis_01658
Aeromonas_caviae_ant3_I
Polynesia_massiliensis_01429
Alkalihalobacillus_clausii_ant4_Ib
Lactobacillus_reuteri_ant4_Ia
Bacilli_ant9
Serratia_marcescens_ant3_Ih
Bacteria_ant
plasmid_in_Staphylococcus_aureus_ant4_Ia
Brevibacillus_brevis_ant4_Ic
plasmid_in_uncultured_bacterium_ant2_Ia
Brevibacillus_brevis_ant6_Ic
Lactobacillus_caccae_00684
Burkholderia_cepacia_ant2_Ia
Traorella_massiliensis_01212
Campylobacter_fetus_ant6_Ib
Anaerococcus_mediannikovii_00814
Citrobacter_freundii_ant2_Ia
Paenibacillus_polymyxa_ant6
Corynebacterium_amycolatum_ant2_Ia
Virgibacillus_dakarensis_04689
Enterobacter_hormaechei_ant1_2
Virgibacillus_massiliensis_02733
Enterococcus_faecium_ant6_Ia
plasmid_in_Enterococcus_faecalis_ant6_Ia
Oceanibacillus_massiliensis_02021
Escherichia_coli_ant2_Ia

Figure S13

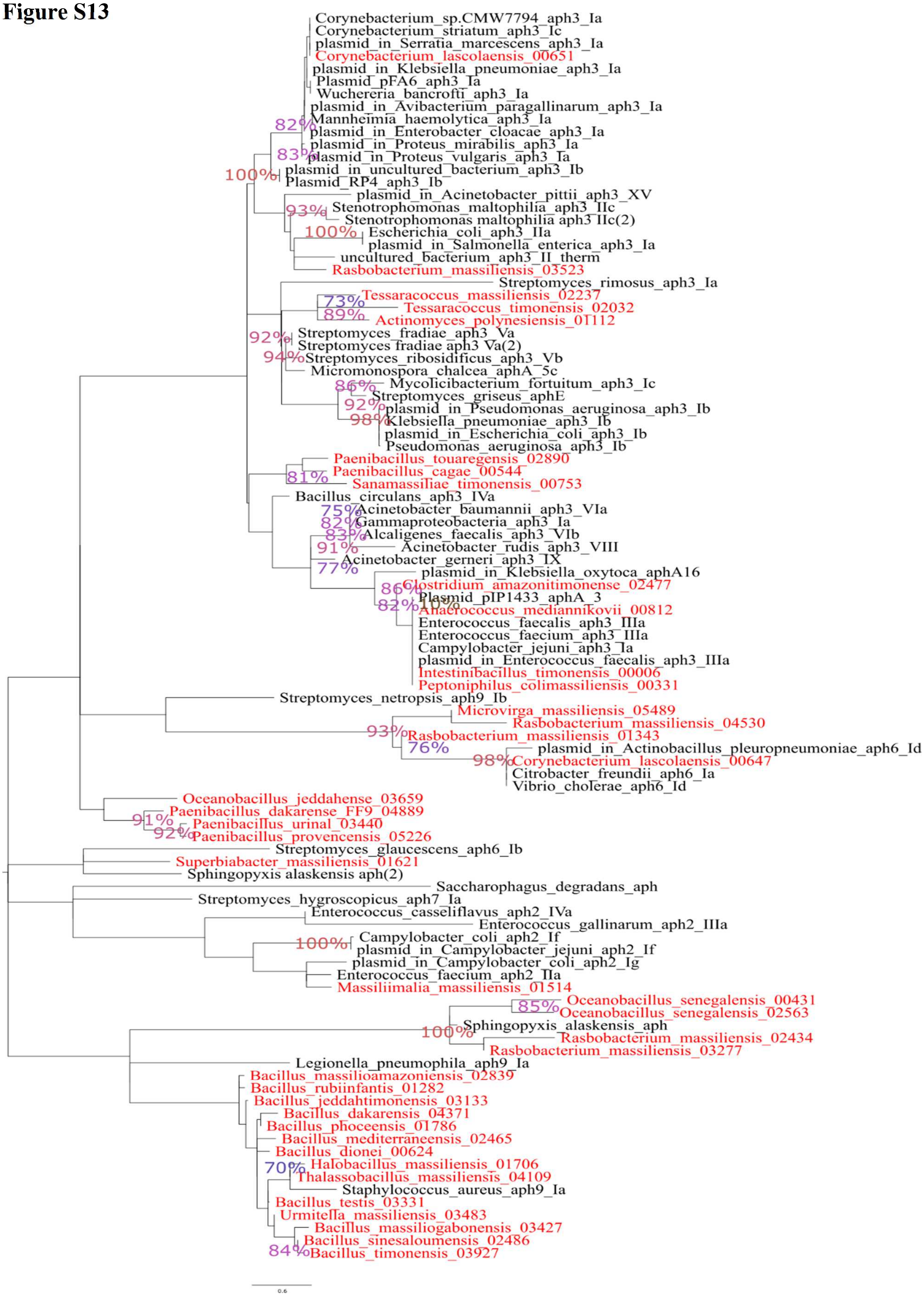
Corynebacterium_sp.CMW7794_aph3_Ia
Corynebacterium_striatum_aph3_Ic
plasmid_in_Serratia_marcescens_aph3_Ia
Corynebacterium_lascolaensis_00651
plasmid_in_Klebsiella_pneumoniae_aph3_Ia
Plasmid_pFA6_aph3_Ia
Wuchereria_bancrofti_aph3_Ia
plasmid_in_Avibacterium_paragallinarum_aph3_Ia
Mannheimia_haemolytica_aph3_Ia
plasmid_in_Enterobacter_cloacae_aph3_Ia
plasmid_in_Proteus_mirabilis_aph3_Ia
plasmid_in_Proteus_vulgaris_aph3_Ia
plasmid_in_uncultured_bacterium_aph3_Ib
Plasmid_RP4_aph3_Ib
plasmid_in_Acinetobacter_pittii_aph3_XV
Stenotrophomonas_maltophilia_aph3_IIc
Stenotrophomonas_maltophilia_aph3_IIc(2)
Escherichia_coli_aph3_IIa
plasmid_in_Salmonella_enterica_aph3_Ia
uncultured_bacterium_aph3_II_therm
Rasbobacterium_massiliensis_03523
Streptomyces_rimosus_aph3_Ia
Tessaracoccus_massiliensis_02237
Tessaracoccus_timonensis_02032
Actinomyces_polynesiensis_01112
Streptomyces_fradiae_aph3_Va
Streptomyces_fradiae_aph3_Va(2)
Streptomyces_ribosidificus_aph3_Vb
Micromonospora_chalcea_aphA_5c
Mycolicibacterium_fortuitum_aph3_Ic
Streptomyces_griseus_aphE
plasmid_in_Pseudomonas_aeruginosa_aph3_Ib
Klebsiella_pneumoniae_aph3_Ib
plasmid_in_Escherichia_coli_aph3_Ib
Pseudomonas_aeruginosa_aph3_Ib
Paenibacillus_touaregensis_02890
Paenibacillus_cagae_00544
Sanamassiliae_timonensis_00753
Bacillus_circulans_aph3_IVa
Acinetobacter_baumannii_aph3_VIa
Gammaproteobacteria_aph3_Ia
Alcaligenes_faecalis_aph3_VIb
Acinetobacter_rudis_aph3_VIII
Acinetobacter_gerneri_aph3_IX
plasmid_in_Klebsiella_oxytoca_aphA16
Clostridium_amazonitimonense_02477
Plasmid_pIP1433_aphA_3
Anaerococcus_mediannikovii_00812
Enterococcus_faecalis_aph3_IIIa
Enterococcus_faecium_aph3_IIIa
Campylobacter_jejuni_aph3_Ia
plasmid_in_Enterococcus_faecalis_aph3_IIIa
Intestinibacillus_timonensis_00006
Peptoniphilus_colimassiliensis_00331
Streptomyces_netropsis_aph9_Ib
Microvirga_massiliensis_05489
Rasbobacterium_massiliensis_04530
Rasbobacterium_massiliensis_01343
plasmid_in_Actinobacillus_pleuropneumoniae_aph6_Id
Corynebacterium_lascolaensis_00647
Citrobacter_freundii_aph6_Ia
Vibrio_cholerae_aph6_Id
Oceanobacillus_jeddahense_03659
Paenibacillus_dakarense_FF9_04889
Paenibacillus_urinal_03440
Paenibacillus_provencensis_05226
Streptomyces_glaucescens_aph6_Ib
Superbiabacter_massiliensis_01621
Sphingopyxis alaskensis aph(2)
Saccharophagus_degradans_aph
Streptomyces_hygroscopicus_aph7_Ia
Enterococcus_casseliflavus_aph2_IVa
Enterococcus_gallinarum_aph2_IIIa
Campylobacter_coli_aph2_If
plasmid_in_Campylobacter_jejuni_aph2_If
plasmid_in_Campylobacter_coli_aph2_Ig
Enterococcus_faecium_aph2_IIa
Massiliimalia_massiliensis_01514
Oceanobacillus_senegalensis_00431
Oceanobacillus_senegalensis_02563
Sphingopyxis_alaskensis_aph
Rasbobacterium_massiliensis_02434
Rasbobacterium_massiliensis_03277
Legionella_pneumophila_aph9_Ia
Bacillus_massilioamazoniensis_02839
Bacillus_rubiinfantis_01282
Bacillus_jeddahtimonensis_03133
Bacillus_dakarensis_04371
Bacillus_phoceensis_01786
Bacillus_mediterraneensis_02465
Bacillus_dionei_00624
Halobacillus_massiliensis_01706
Thalassobacillus_massiliensis_04109
Staphylococcus_aureus_aph9_Ia
Bacillus_testis_03331
Urmitella_massiliensis_03483
Bacillus_massiliogabonensis_03427
Bacillus_sinesaloumensis_02486
Bacillus_timonensis_03927
82%
83%
100%
93%
100%
73%
89%
92%
94%
86%
92%
98%
81%
75%
82%
83%
91%
77%
86%
82%
93%
76%
98%
91%
92%
100%
85%
100%
70%
84%
0.6

Figure S15

Figure S14

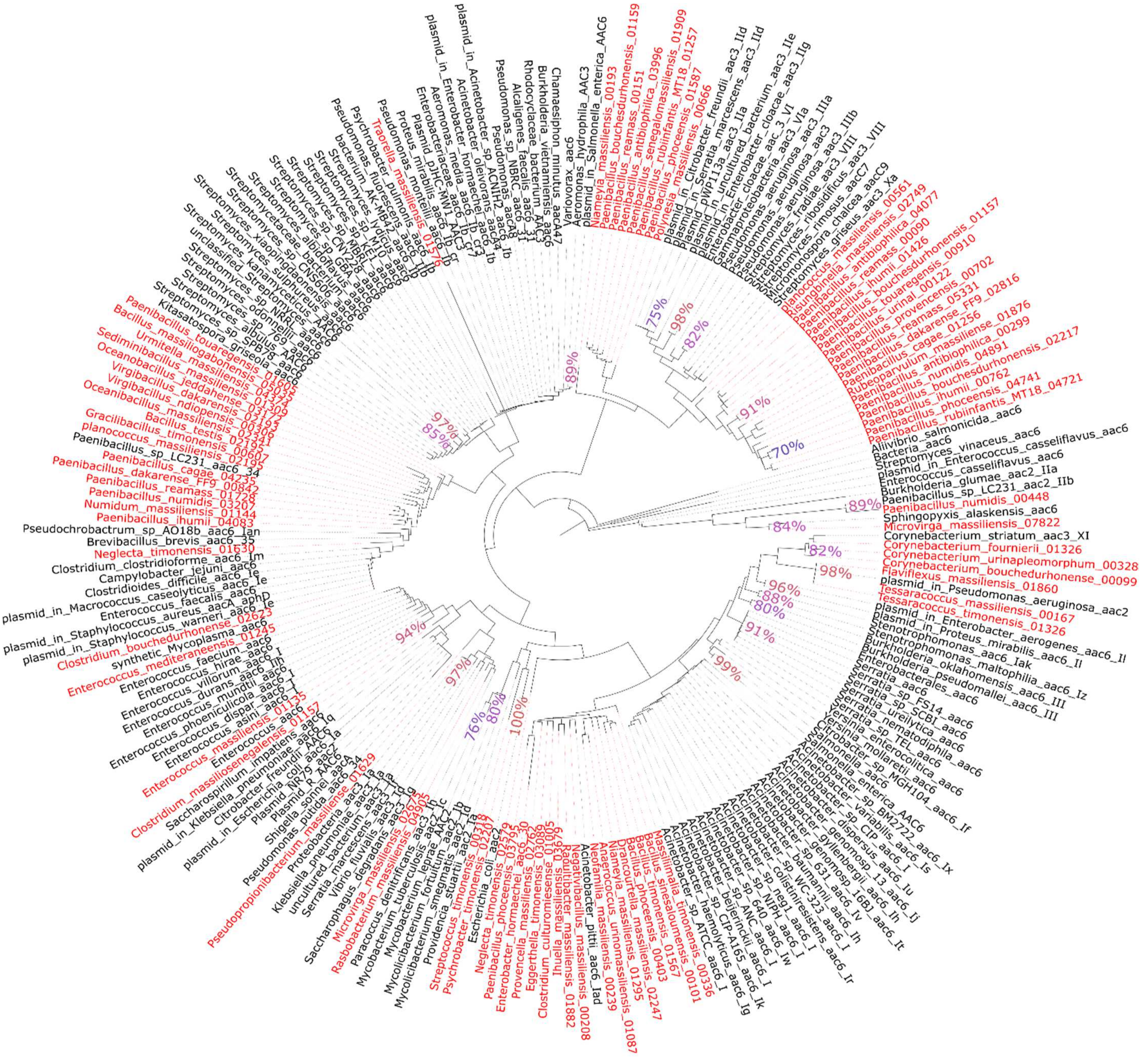

Figure S15

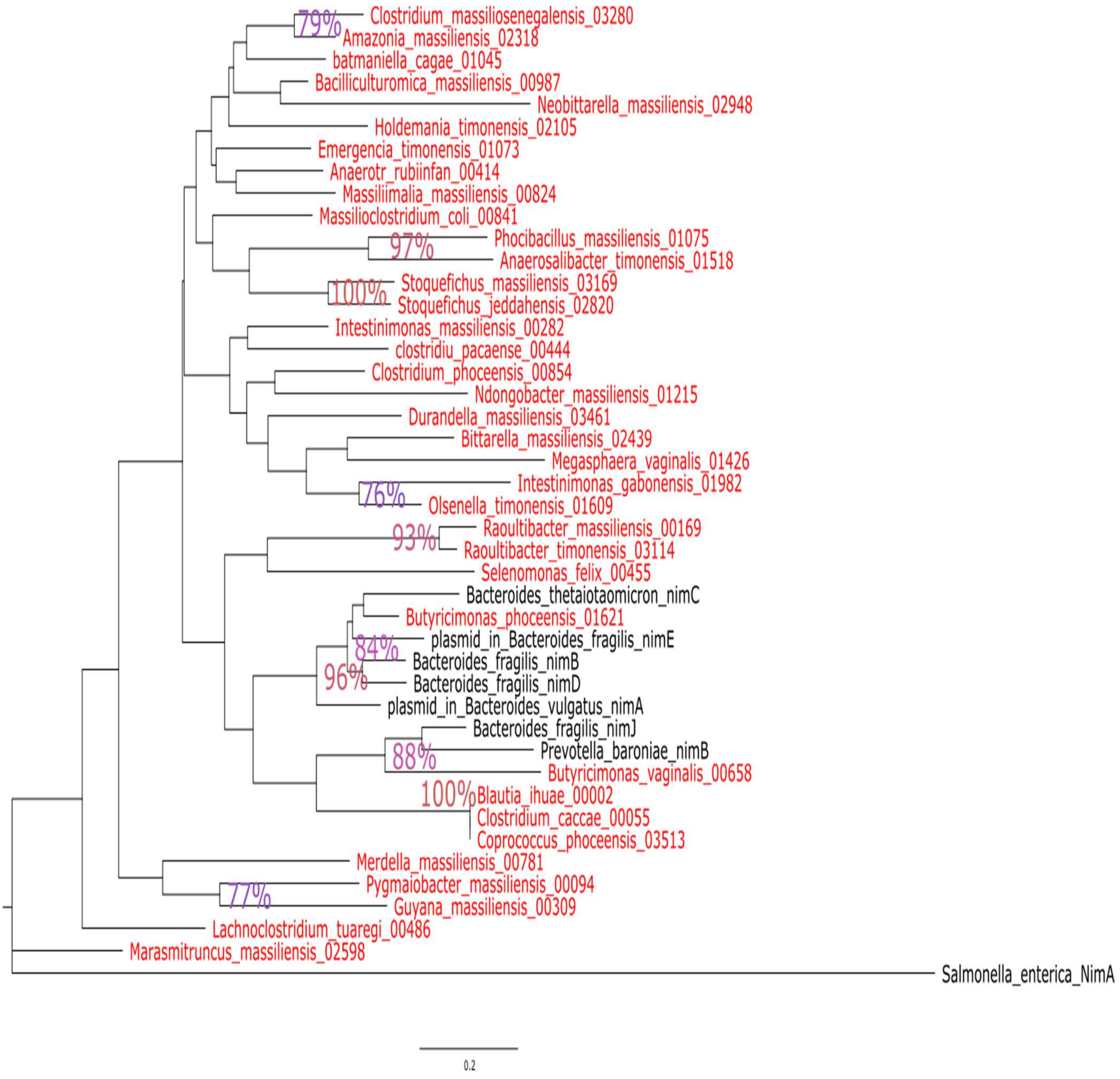
Clostridium_massiliosenegalensis_03280
79%
Amazonia_massiliensis_02318
batmaniella_cagae_01045
Bacilliculturomica_massiliensis_00987
Neobittarella_massiliensis_02948
Holdemania_timonensis_02105
Emergencia_timonensis_01073
Anaerotr_rubiinfan_00414
Massiliimalia_massiliensis_00824
Massilioclostridium_coli_00841
Phocibacillus_massiliensis_01075
97%
Anaerosalibacter_timonensis_01518
Stoquefichus_massiliensis_03169
100%
Stoquefichus_jeddahensis_02820
Intestinimonas_massiliensis_00282
clostridiu_pacaense_00444
Clostridium_phoceensis_00854
Ndongobacter_massiliensis_01215
Durandella_massiliensis_03461
Bittarella_massiliensis_02439
Megasphaera_vaginalis_01426
Intestinimonas_gabonensis_01982
76%
Olsenella_timonensis_01609
Raoultibacter_massiliensis_00169
93%
Raoultibacter_timonensis_03114
Selenomonas_felix_00455
Bacteroides_thetaiotaomicron_nimC
Butyricimonas_phoceensis_01621
plasmid_in_Bacteroides_fragilis_nimE
84%
Bacteroides_fragilis_nimB
96%
Bacteroides_fragilis_nimD
plasmid_in_Bacteroides_vulgatus_nimA
Bacteroides_fragilis_nimJ
Prevotella_baroniae_nimB
88%
Butyricimonas_vaginalis_00658
100%
Blautia_ihuae_00002
Clostridium_caccae_00055
Coprococcus_phoceensis_03513
Merdella_massiliensis_00781
Pygmaiobacter_massiliensis_00094
77%
Guyana_massiliensis_00309
Lachnoclostridium_tuaregi_00486
Marasmitruncus_massiliensis_02598
Salmonella_enterica_NimA
0.2

Figure S16

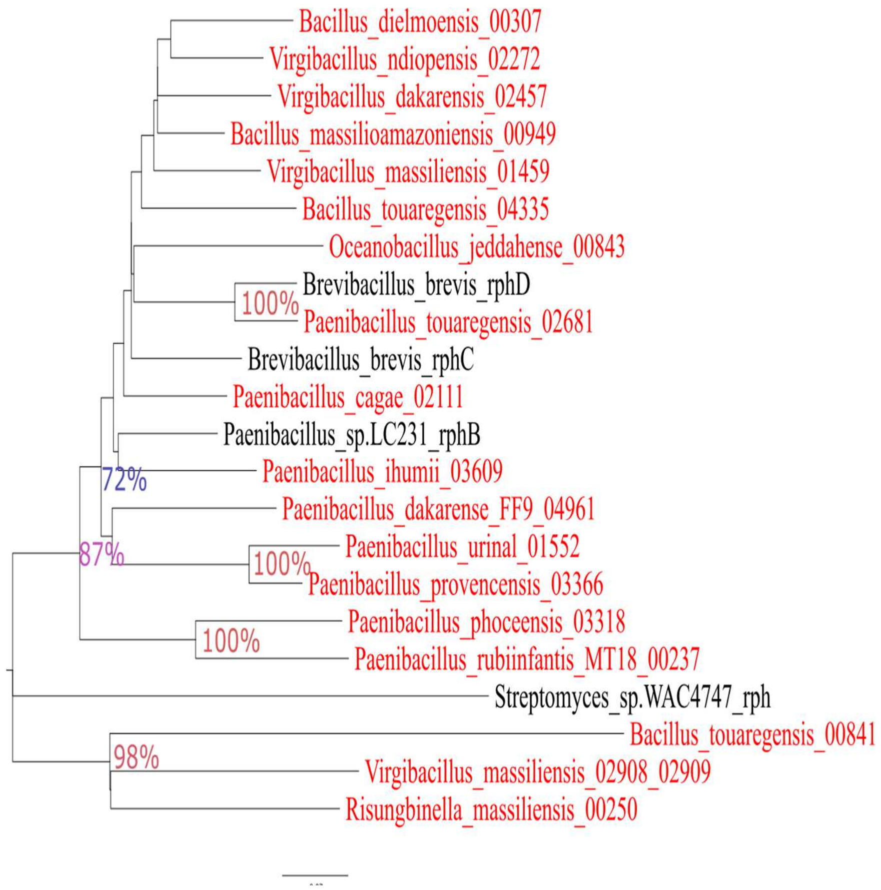

Figure S17

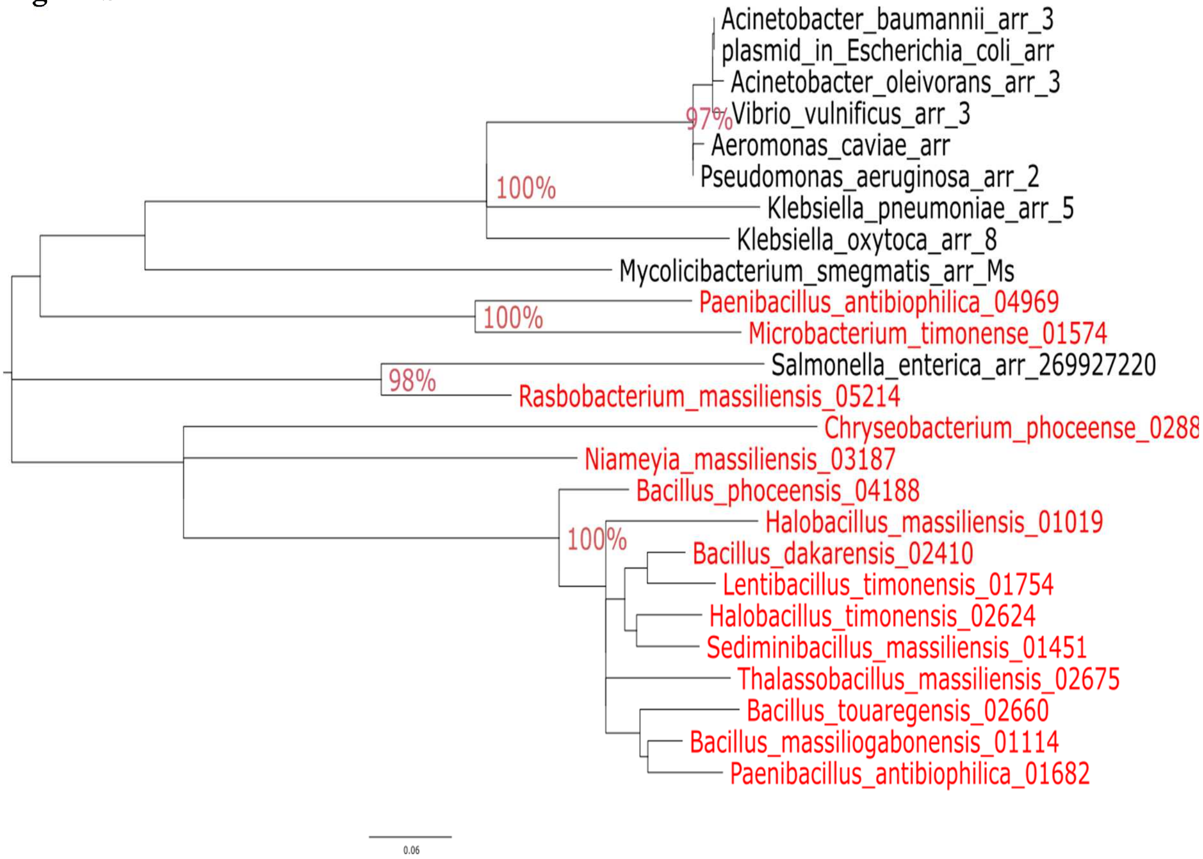

Figure S18

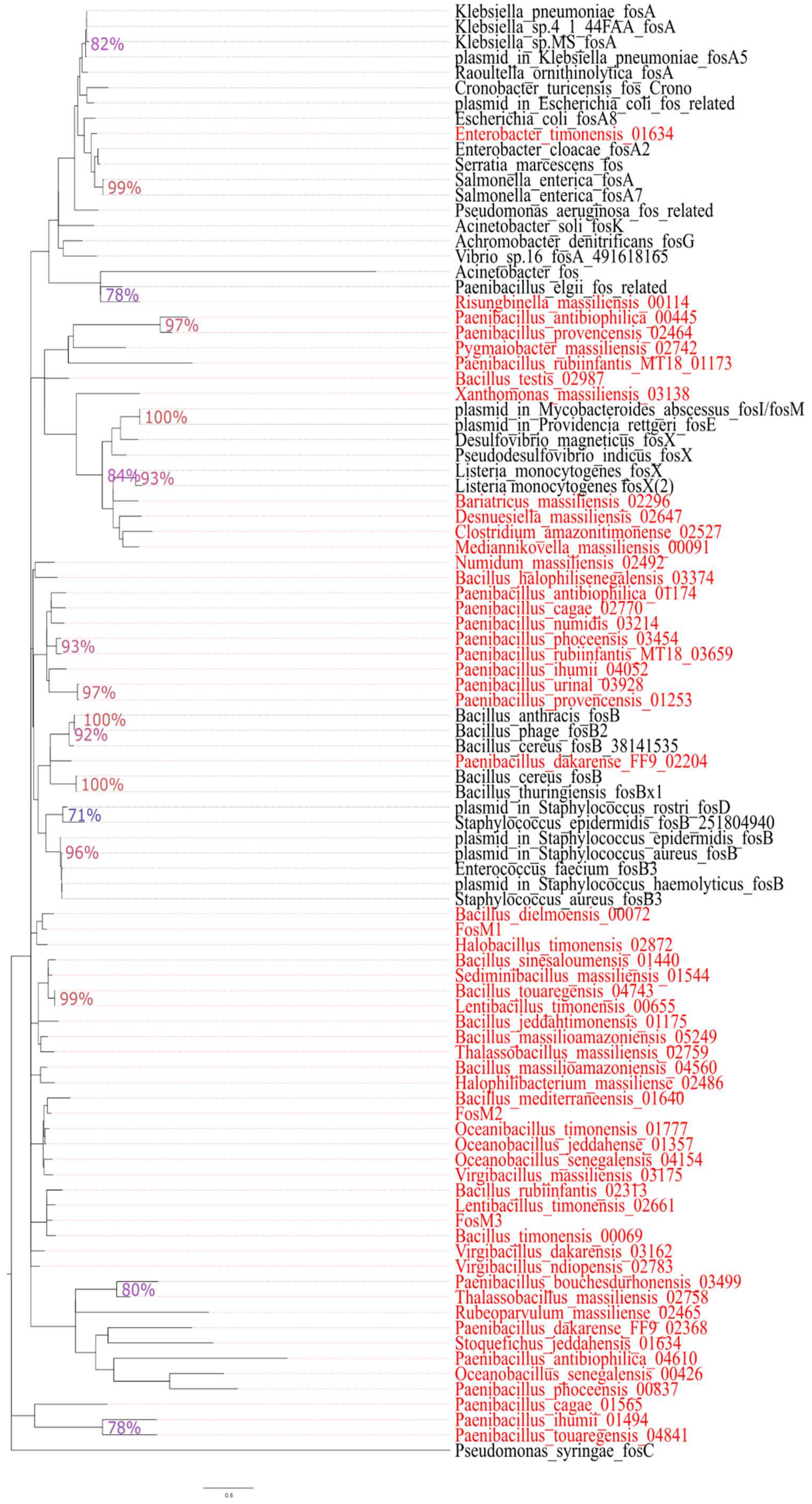

Klebsiella_pneumoniae_fosA
Klebsiella_sp.4_1_44FAA_fosA
Klebsiella_sp.MS_fosA
plasmid_in_Klebsiella_pneumoniae_fosA5
Raoultella_ornithinolytica_fosA
Cronobacter_turicensis_fos_Crono
plasmid_in_Escherichia_coli_fos_related
Escherichia_coli_fosA8
Enterobacter_timonensis_01634
Enterobacter_cloacae_fosA2
Serratia_marcescens_fos
Salmonella_enterica_fosA
Salmonella_enterica_fosA7
Pseudomonas_aeruginosa_fos_related
Acinetobacter_soli_fosK
Achromobacter_denitrificans_fosG
Vibrio_sp.16_fosA_491618165
Acinetobacter_fos
Paenibacillus_elgii_fos_related
Risungbinella_massiliensis_00114
Paenibacillus_antibiophilica_00445
Paenibacillus_provencensis_02464
Pygmaiobacter_massiliensis_02742
Paenibacillus_rubiinfantis_MT18_01173
Bacillus_testis_02987
Xanthomonas_massiliensis_03138
plasmid_in_Mycobacteroides_abscessus_fosI/fosM
plasmid_in_Providencia_rettgeri_fosE
Desulfovibrio_magneticus_fosX
Pseudodesulfovibrio_indicus_fosX
Listeria_monocytogenes_fosX
Listeria_monocytogenes_fosX(2)
Bariatricus_massiliensis_02296
Desnuesiella_massiliensis_02647
Clostridium_amazonitimonense_02527
Mediannikovella_massiliensis_00091
Numidum_massiliensis_02492
Bacillus_halophilisenegalensis_03374
Paenibacillus_antibiophilica_01174
Paenibacillus_cagae_02770
Paenibacillus_numidis_03214
Paenibacillus_phoceensis_03454
Paenibacillus_rubiinfantis_MT18_03659
Paenibacillus_ihumii_04052
Paenibacillus_urinal_03928
Paenibacillus_provencensis_01253
Bacillus_anthracis_fosB
Bacillus_phage_fosB2
Bacillus_cereus_fosB_38141535
Paenibacillus_dakarense_FF9_02204
Bacillus_cereus_fosB
Bacillus_thuringiensis_fosBx1
plasmid_in_Staphylococcus_rostri_fosD
Staphylococcus_epidermidis_fosB_251804940
plasmid_in_Staphylococcus_epidermidis_fosB
plasmid_in_Staphylococcus_aureus_fosB
Enterococcus_faecium_fosB3
plasmid_in_Staphylococcus_haemolyticus_fosB
Staphylococcus_aureus_fosB3
Bacillus_dielmoensis_00072
FosM1
Halobacillus_timonensis_02872
Bacillus_sinesaloumensis_01440
Sediminibacillus_massiliensis_01544
Bacillus_touaregensis_04743
Lentibacillus_timonensis_00655
Bacillus_jeddahtimonensis_01175
Bacillus_massilioamazoniensis_05249
Thalassobacillus_massiliensis_02759
Bacillus_massilioamazoniensis_04560
Halophilibacterium_massiliense_02486
Bacillus_mediterraneensis_01640
FosM2
Oceanibacillus_timonensis_01777
Oceanobacillus_jeddahense_01357
Oceanobacillus_senegalensis_04154
Virgibacillus_massiliensis_03175
Bacillus_rubiinfantis_02313
Lentibacillus_timonensis_02661
FosM3
Bacillus_timonensis_00069
Virgibacillus_dakarensis_03162
Virgibacillus_ndiopensis_02783
Paenibacillus_bouchesdurhonensis_03499
Thalassobacillus_massiliensis_02758
Rubeoparvulum_massiliense_02465
Paenibacillus_dakarense_FF9_02368
Stoquefichus_jeddahensis_01634
Paenibacillus_antibiophilica_04610
Oceanobacillus_senegalensis_00426
Paenibacillus_phoceensis_00837
Paenibacillus_cagae_01565
Paenibacillus_ihumii_01494
Paenibacillus_touaregensis_04841
Pseudomonas_syringae_fosC
82%
99%
78%
97%
100%
84%
93%
93%
97%
100%
92%
100%
71%
96%
99%
80%
78%
0.6

Figure S19

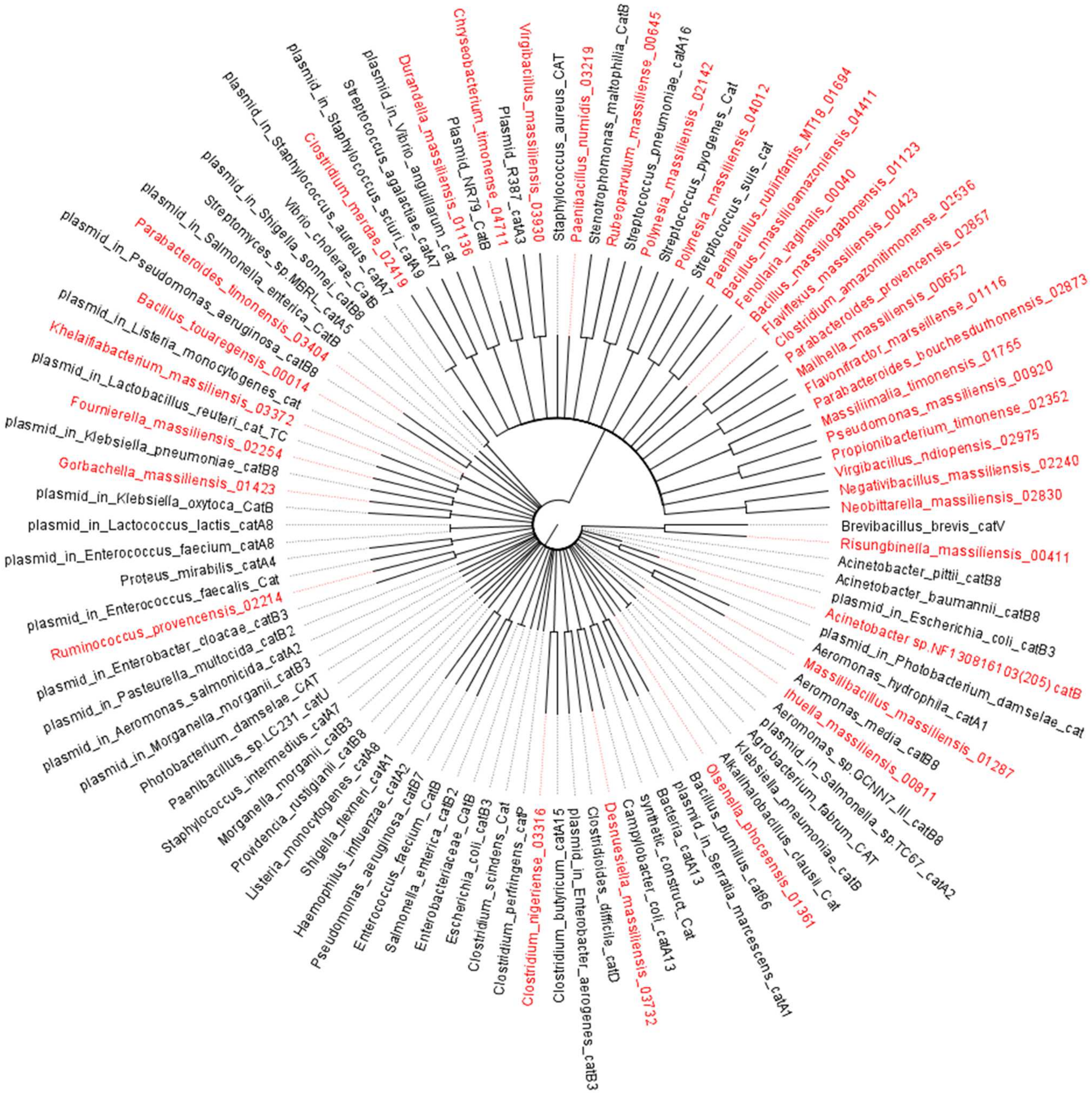
Brevibacillus_brevis_catV
Risungbinella_massiliensis_00411
Acinetobacter_pittii_catB8
Acinetobacter_baumannii_catB8
plasmid_in_Escherichia_coli_catB3
Acinetobacter sp.NF130816103(205) catB
plasmid_in_Photobacterium_damselae_cat
Aeromonas_hydrophila_catA1
Massilibacillus_massiliensis_01287
Aeromonas_media_catB8
Ihuella_massiliensis_00811
Aeromonas_sp.GCNN7_III_catB8
plasmid_in_Salmonella_sp.TC67_catA2
Agrobacterium_fabrum_CAT
Klebsiella_pneumoniae_catB
Alkalihalobacillus_clausii_Cat
Olsenella_phoceensis_01361
Bacillus_pumilus_cat86
plasmid_in_Serratia_marcescens_catA1
Bacteria_catA13
synthetic_construct_Cat
Campylobacter_coli_catA13
Desnuesiella_massiliensis_03732
Clostridioides_difficile_catD
plasmid_in_Enterobacter_aerogenes_catB3
Clostridium_butyricum_catA15
Clostridium_nigeriense_03316
Clostridium_perfringens_catP
Clostridium_scindens_Cat
Escherichia_coli_catB3
Enterobacteriaceae_CatB
Salmonella_enterica_catB2
Enterococcus_faecium_CatB
Pseudomonas_aeruginosa_catB7
Haemophilus_influenzae_catA2
Shigella_flexneri_catA1
Listeria_monocytogenes_catA8
Providencia_rustigianii_catB3
Morganella_morganii_catB8
Staphylococcus_intermedius_catA7
Paenibacillus_sp.LC231_catU
Photobacterium_damselae_CAT
plasmid_in_Morganella_morganii_catB3
plasmid_in_Aeromonas_salmonicida_catA2
plasmid_in_Pasteurella_multocida_catB2
plasmid_in_Enterobacter_cloacae_catB3
Ruminococcus_provencensis_02214
plasmid_in_Enterococcus_faecalis_Cat
Proteus_mirabilis_catA4
plasmid_in_Enterococcus_faecium_catA8
plasmid_in_Lactococcus_lactis_catA8
plasmid_in_Klebsiella_oxytoca_CatB
Gorbachella_massiliensis_01423
plasmid_in_Klebsiella_pneumoniae_catB8
Fournierella_massiliensis_02254
plasmid_in_Lactobacillus_reuteri_cat_TC
Khelaifiabacterium_massiliensis_03372
plasmid_in_Listeria_monocytogenes_cat
Bacillus_touaregensis_00014
plasmid_in_Pseudomonas_aeruginosa_catB8
Parabacteroides_timonensis_03404
plasmid_in_Salmonella_enterica_CatB
Streptomyces_sp.MBRL_catA5
plasmid_in_Shigella_sonnei_catB8
Vibrio_cholerae_CatB
plasmid_in_Staphylococcus_aureus_catA7
Clostridium_merdae_02419
plasmid_in_Staphylococcus_sciuri_catA9
Streptococcus_agalactiae_catA7
plasmid_in_Vibrio_anguillarum_cat
Durandella_massiliensis_01136
Plasmid_NR79_CatB
Chryseobacterium_timonense_04711
Plasmid_R387_catA3
Virgibacillus_massiliensis_03930
Staphylococcus_aureus_CAT
Paenibacillus_numidis_03219
Stenotrophomonas_maltophilia_CatB
Rubeoparvulum_massiliense_00645
Streptococcus_pneumoniae_catA16
Polynesia_massiliensis_02142
Streptococcus_pyogenes_Cat
Polynesia_massiliensis_04012
Streptococcus_suis_cat
Paenibacillus_rubinfantis_MT18_01694
Bacillus_massilioamazoniensis_04411
Fenollaria_vaginalis_00040
Bacillus_massiliogabonensis_01123
Flaviflexus_massiliensis_00423
Clostridium_amazonitimonense_02536
Parabacteroides_provencensis_02857
Mailhella_massiliensis_00652
Flavonifractor_marseillense_01116
Parabacteroides_bouchesdurhonensis_02873
Massiliimalia_timonensis_01755
Pseudomonas_massiliensis_00920
Propionibacterium_timonense_02352
Virgibacillus_ndiopensis_02975
Negativibacillus_massiliensis_02240
Neobittarella_massiliensis_02830
